# Reading Minds, Reading Stories: Social-Cognitive Abilities Affect the Linguistic Processing of Narrative Viewpoint

**DOI:** 10.3389/fpsyg.2021.698986

**Published:** 2021-09-28

**Authors:** Lynn S. Eekhof, Kobie van Krieken, José Sanders, Roel M. Willems

**Affiliations:** ^1^Centre for Language Studies, Radboud University, Nijmegen, Netherlands; ^2^Donders Institute for Brain, Cognition, and Behaviour, Radboud University, Nijmegen, Netherlands; ^3^Max Planck Institute for Psycholinguistics, Nijmegen, Netherlands

**Keywords:** social cognition, narrative, viewpoint, perspective, eye tracking

## Abstract

Although various studies have shown that narrative reading draws on social-cognitive abilities, not much is known about the precise aspects of narrative processing that engage these abilities. We hypothesized that the linguistic processing of narrative viewpoint—expressed by elements that provide access to the inner world of characters—might play an important role in engaging social-cognitive abilities. Using eye tracking, we studied the effect of lexical markers of perceptual, cognitive, and emotional viewpoint on eye movements during reading of a 5,000-word narrative. Next, we investigated how this relationship was modulated by individual differences in social-cognitive abilities. Our results show diverging patterns of eye movements for perceptual viewpoint markers on the one hand, and cognitive and emotional viewpoint markers on the other. Whereas the former are processed relatively fast compared to non-viewpoint markers, the latter are processed relatively slow. Moreover, we found that social-cognitive abilities impacted the processing of words in general, and of perceptual and cognitive viewpoint markers in particular, such that both perspective-taking abilities and self-reported perspective-taking traits facilitated the processing of these markers. All in all, our study extends earlier findings that social cognition is of importance for story reading, showing that individual differences in social-cognitive abilities are related to the linguistic processing of narrative viewpoint.

## Introduction

Although reading might seem a rather solitary activity compared to engaging in social interaction, many scholars have argued that social-cognitive processes play an important role during story reading. That is, the abilities we use in our daily lives to make sense of the emotions, beliefs, intentions, and behavior of others, such as empathy, emotion recognition, and theory of mind, are also engaged when we read about fictional others in stories (Oatley, 1999; Zunshine, 2006; Mar and Oatley, 2008). However, despite research underlining the importance of social-cognitive abilities for story reading, it is not clear exactly what aspects of narrative processing require readers to put these abilities to work. In other words, there is relatively little research on the relationship between social-cognitive abilities and the processing of specific narrative characteristics.

In this study we therefore investigated how individual differences in readers' social-cognitive abilities are related to a crucial aspect of narrative processing, namely the linguistic processing of narrative viewpoint. In what follows, we will first discuss the role of social cognition during narrative reading. After introducing the multidimensional concept of narrative viewpoint, we will discuss why the linguistic processing of narrative viewpoint is likely related to readers' social-cognitive abilities.

### Social Cognition and Narrative Reading

The contention that narratives engage social-cognitive abilities follows from two views on what constitutes a narrative. Firstly, narratives are often loosely defined as the representation of a sequence of events that are related in time (e.g., Toolan, 2001; for an overview, see Ryan, 2007; Abbott, 2008). In line with these plot-focused definitions, Zunshine (2003, 2006) has argued that much like displays of behavior in real life, textual descriptions of narrative events can invite readers to use their theory of mind abilities to assign mental states to the characters performing these events. For example, descriptions of the actions and/or body language of characters might leave the reader wondering why a character behaves in a certain way, or guessing how the character feels, living through these events. Hence, on this account, social cognition might play an important role in making sense of the plot of narratives.

In addition, scholars have stressed the subjective aspect of narratives (e.g., Bruner, 1986; Bal, 2009). For example, Bal (2009, p. 10) gives the following definition: “[…] a series of connected events caused or *experienced by actors* presented in a specific manner” (emphasis added). On such accounts, narratives are unique because the events always presuppose the presence of someone who experiences these events. As a result, authors can choose to directly represent the internal states of their protagonist through the use of, for example, mental verbs (*to think, to believe*) or other perspectivization techniques that grant the reader direct access to the mind of story characters (van Krieken et al., 2017; Eekhof et al., 2020). These mental representations might also elicit a form of perspective taking in readers (van Krieken et al., 2017).

Comprising the above approaches, narratives can be seen as a sequence of textual cues, guiding the reader to form a cognitive, social, and emotional simulation of what is described in the narrative (Oatley, 1999; Mar and Oatley, 2008). Crucially, such a simulation also requires readers to employ social-cognitive abilities to “reconstruct the minds” of the narrative characters (Ryan, 2007, p. 28). In a similar vein, Koopman and Hakemulder (2015, p. 91) argue that an important aspect of being absorbed in a story world is “empathic imagination,” a process whereby the reader uses empathic abilities to imagine “how it would be to be in the shoes of a particular character.”

Several studies provide (indirect) evidence for the involvement of social-cognitive abilities during narrative reading. For example, a range of fMRI studies has shown that brain regions that are part of the mentalizing network (e.g., mPFC, bilateral pSTS/TPJ) are also activated during narrative comprehension (e.g., Mason and Just, 2009; for a meta-analysis see Mar, 2011). Furthermore, theory of mind development in children parallels developments in the processing of narratives. For example, recall of socially relevant details of a story has been found to increase between adolescence and adulthood, potentially mirroring a development in social-cognitive abilities in the same period (Pavias et al., 2016). Similarly, in a story retelling task, both age and theory of mind abilities were found to positively predict the ability to coordinate story characters' actions and mental states in preschoolers (Pelletier and Wilde Astington, 2004; see also Fernández, 2013). Finally, in a longitudinal study, children's theory of mind scores at age four predicted narrative comprehension and recall two and a half years later (Atkinson et al., 2017). Taken together, these studies tentatively suggest that social-cognitive abilities play a role in narrative comprehension, both in adults and in children.

However, many of the previous studies have taken a rather broad look at narrative processing, looking at the relationship between social cognition and story reading in general (fMRI studies), or narrative comprehension and recall after reading (developmental studies). As a result, not much in known yet about the specific aspects of narrative processing that engage readers' social-cognitive abilities. Two fMRI studies, however, did find that processing stories rich in descriptions of characters' mental states (Tamir et al., 2016) and stories with negative valence (i.e., dealing with negative events such as crimes and disasters; Altmann et al., 2012) elicited more activation in brain regions related to theory of mind (e.g., dmPFC subnetwork) compared to stories with less socially relevant content and stories with positive valence, suggesting that, broadly speaking, processing of social and negatively valenced narrative content draws on social-cognitive abilities. Nevertheless, more research is needed to elucidate what exactly it is about narratives that requires readers to use their social-cognitive abilities. That is, future studies should provide a more detailed account of the facets of narrative processing that engage social cognition.

### Narrative Viewpoint

An aspect of narrative processing that might play a role in the engagement of social-cognitive abilities during reading is the linguistic processing of narrative viewpoint. As explained above, narratives presuppose the presence of an “experiencing subject” (Sanders and Redeker, 1996). Typically, the events in narratives are always grounded in and related through the subjective viewpoints (or perspectives) of these experiencing characters and/or narrators (Sanders, 1994). During reading, readers align themselves with the events and dynamically take the perspective of one or more of the characters and/or narrators, both in terms of their spatio-temporal viewpoint in the narrative world and in terms of their inner viewpoint or consciousness (Vandelanotte, 2017). In their Linguistic Cues Framework, van Krieken et al. (2017) distinguish between multiple dimensions of viewpoint and argue that each dimension is regulated by different linguistic cues. For example, perceptual viewpoint, referring to the narrative representation of characters' perceptions and sensations, can be expressed through the use of perceptual verbs (*to watch, to hear*), emotional viewpoint, referring to the narrative representation of characters' emotions, can be expressed through the use of emotion adjectives (*angry, delighted*), and cognitive viewpoint, referring to the narrative representation of characters' mental states, can be expressed through epistemic markers (*probably, definitely*). Crucially, these linguistic viewpoint markers are hypothesized to invite the reader to identify with a particular subject in the narrative (van Krieken et al., 2017). In other words, linguistic markers of viewpoint can be seen as a signal to the reader to engage in perspective taking. As such, viewpoint markers might play an important role in eliciting social-cognitive processes during narrative reading, given that perspective taking is an important aspect of social cognition (Frith and Frith, 2006; Goldman and de Vignemont, 2009; Healey and Grossman, 2018).

Interestingly, literature on the development of language and theory of mind provides evidence that social cognition plays a role in the linguistic processing of viewpoint markers such as verbs of cognition and emotion, although this has not always been tested in narrative contexts (for a general overview on the relationship between language acquisition and theory of mind acquisition see Milligan et al., 2007). For example, comprehension of verbs of cognition in short stories has been found to be related to performance on first-order false belief tasks, and to a lesser degree to second-order false belief tasks in children aged between 4 and 8 years (Antonietti et al., 2006), and to second-order false belief tasks in children aged between 8 and 11 years (Grazzani and Ornaghi, 2012). Similarly, in a task that required children to make sense of spoken instructions to find an object, comprehension of modal verbs and adjectives, which can be considered markers of cognitive viewpoint (van Krieken et al., 2017; Eekhof et al., 2020), was significantly related to performance on first-order false belief tasks in four-year-olds (Moore et al., 1990). Furthermore, comprehension of verbs of emotion on a short-story task was significantly correlated to emotion understanding in a study with seven- to ten-year-olds (Ornaghi and Grazzani, 2013). These results indicate that individual differences in social-cognitive abilities are related to the linguistic processing of at least emotional and cognitive viewpoint markers in children, suggesting that social cognition and the processing of narrative viewpoint somehow go hand in hand.

All in all, viewpoint markers are likely to play a role in engaging social-cognitive processes during the reading of narratives, as at least in childhood the processing of viewpoint markers has been found to be related to individual differences in social-cognitive abilities. Hence, we wanted to further investigate the relationship between the linguistic processing of viewpoint markers in narratives and social-cognitive abilities in adult readers. Our rationale was that if individual differences in social-cognitive abilities affect the linguistic processing of narrative viewpoint, this highly suggests that markers of narrative viewpoint engage these social-cognitive abilities.

### The Current Study

We set out to study how the linguistic processing of narrative viewpoint markers is affected by individual differences in social-cognitive abilities, using eye-tracking. Hence, as a first step we aimed to find out how perceptual, cognitive, and emotional viewpoint markers affect reading behavior. More importantly, we then aimed to study how these effects are modulated by social-cognitive abilities. In sum, the current study aimed to answer the following research question:

*What is the effect of perceptual, cognitive, and emotional viewpoint markers in narratives on reading behavior, and how is this effect modulated by individual differences in social-cognitive abilities?*

Based on a study by Mak and Willems (2018), who found that narrative passages describing characters' perceptions, thoughts, and emotions increased reading times, we hypothesized viewpoint markers to be processed slower than non-viewpoint markers. We also hypothesized that, in general, social-cognitive abilities would modulate the effect of viewpoint markers on reading behavior. More specifically, and based on the research that shows that theory of mind abilities positively predict narrative comprehension in general (e.g., Atkinson et al., 2017), and the acquisition of epistemic markers, verbs of cognition, and verbs of emotion specifically (Moore et al., 1990; Antonietti et al., 2006; Grazzani and Ornaghi, 2012; Ornaghi and Grazzani, 2013), we tentatively hypothesized that social-cognitive abilities lead to faster processing of viewpoint markers (i.e., more skipping, shorter gaze durations, less rereading). We did not have specific hypotheses about the modulating effect of social-cognitive abilities for each specific viewpoint marker category separately.

## Methods

An eye-tracking study was designed to study the linguistic processing of viewpoint markers, by looking at the effect of these markers on skip rate, gaze duration, and re-reading rate (for the justification of these eye-tracking methods, see Pre-Processing of Eye-Tracking Data). We chose to focus on markers of perceptual, cognitive, and emotional viewpoint as we expected the processing of these viewpoint dimensions to be most relevant to the domain of social cognition. We opted for eye tracking as an appropriate method for several reasons. Firstly, contrary to, for example, self-paced reading, eye tracking provides a relatively ecologically valid way to study reading, as participants can be presented with large pieces of texts without any additional task. Furthermore, eye tracking has proven to be a useful method to study individual differences in narrative processing, as evidenced by recent studies on individual differences in mental simulation (Mak and Willems, 2018), sensitivity to literary style (van den Hoven et al., 2016), sensitivity to lexical characteristics and absorption (Eekhof et al., 2021), metaphor processing (Vries et al., 2018), and reading style (Faber et al., 2020) during story reading. Contrary to previous studies, we used a non-fictional rather than a fictional narrative, published in a well-established journalistic weekly magazine. A main function of narrative journalism is to increase the general audience's understanding of society in all its complexities and to enhance the audience's sense of being part of that society (van Krieken and Sanders, 2021). In this genre, narrative perspective taking is typically stimulated by multiple linguistic viewpoints that readers are invited to share (van Krieken et al., 2015). As viewpoint techniques are typical of narratives regardless of their fictionality we believe research on the relationship between social cognition and narratives should be expanded to include non-fictional narratives as well (see also Koopman and Hakemulder, 2015).

### Participants

Based on a power simulation (see Supplementary Materials) we aimed for a sample of 90 participants. Taking into account the high rate of data rejection in eye-tracking studies with long texts, we recruited 114 native speakers of Dutch with normal or corrected to normal vision and no history of reading disorders from the participant pool of Radboud University to take part in the experiment in exchange for money (€15) or course credit. Three participants did not finish the experiment because of time constraints or technical failure. Of the remaining participants, 21 had to be excluded due to poor quality of eye-tracking data (see Pre-Processing of Eye-Tracking Data). After data rejection, the final sample contained data from 90 participants, aged between 18 and 48 (*M* = 23.30, *SD* = 5.49, 67 females, 23 males). The experiment was conducted in accordance with the declaration of Helsinki and was approved by the institutional ethics assessment committee (Approval Number 2018-3568).

### Materials

#### Narrative

A Dutch non-fictional narrative (i.e., journalistic narrative; see van Krieken, 2019; van Krieken and Sanders, 2021) published in a weekly Dutch news magazine, *Vrij Nederland*, was presented to all participants^1^. The story describes a real-life missing person case and is told from the perspective of the missing man's brother, who struggles to find peace during the 16 years that his younger brother is missing. At the end of the story, the missing man's remains are found in a river and it is revealed that he has passed away as the result of a car crash. All paratextual elements (e.g., pictures and pull quotes) except the title were removed for the experiment, resulting in a 5,077-word text^2^.

The ViewPoint Identification Procedure (VPIP; Eekhof et al., 2020) was applied to identify all markers of perceptual, cognitive, and emotional viewpoint in the narrative. This procedure defines perceptual viewpoint markers as content words that express the perceptual viewpoint, i.e., the perceptions and bodily sensations, of characters and/or narrators, and operationalizes these as verbs of perception (e.g., *to see, to hear, to smell*), verbs of bodily sensation (e.g., *to itch, to sting*), and other content words morphologically related to these verb types (e.g., *sight-to see, itchy-to itch*). Cognitive viewpoint markers are defined as content words that express the cognitive viewpoint, i.e., the thoughts, beliefs, intentions and/or desires, of characters and/or narrators. These markers are operationalized as verbs of cognition (e.g., *to think, to believe*), including modal epistemic verbs (e.g., *should, might*), modal epistemic adverbs (e.g., *possibly, definitely*), and morphologically related content words (e.g., *thought-to think, possible-possibly*). Finally, the VPIP defines emotional viewpoint markers as content words that express the emotional viewpoint, i.e., the emotions, of characters and/or narrators, and operationalizes these as verbs of emotion (e.g., *to disdain, to love*), adjectives of emotion (e.g., *angry, bewildered*), and morphologically related content words (e.g., *disdain-to disdain, anger-angry*).

The narrative was coded by the first author according to the steps of the VPIP (Eekhof et al., 2020). That is, first the text was read, then the narrative was divided into 5,032 lexical units, with complex phrasal verbs (e.g., *uitkijken, hij kijkt uit* “to look out, he looks out”) being treated as a single lexical unit. Function words were then disregarded, and for the remaining content words it was determined whether the lexical unit in its narrative context was related to one of the three viewpoint dimensions, and whether the lexical unit could be considered a viewpoint marker for that dimension. To assess the reliability of the procedure, 20% of the content words of the narrative were then also independently coded by the second author. As inter-rater reliability for both the binary decision (viewpoint marker vs. non-viewpoint marker; 96.81%, κ = 0.84), and categorical decision (perceptual vs. cognitive vs. emotional vs. non-viewpoint markers; 96.31%, κ = 0.82) were almost perfect, the ratings of the first author were used for the analyses. Two hundred and ninty-two lexical units (300 words) were scored as viewpoint markers: 86 lexical units (93 words) were marked as perceptual viewpoint markers, 146 lexical units (148 words) were marked as cognitive viewpoint markers, and 59 lexical units (59 words) were marked as emotional viewpoint markers. An example from the coded narrative is given in Table 1. More examples can be found in Supplementary Table 1. All words that were not coded as perceptual, cognitive, or emotional viewpoint markers, were marked as “non-viewpoint marker.” As the viewpoint markers were all content words, we decided to also disregard function words from the non-viewpoint marker category. Hence, besides the 300 viewpoint markers, 2,510 non-viewpoint marking content words were used as a baseline in the analyses (see also Pre-Processing of Eye-Tracking Data). For information on the distribution of word classes in the different viewpoint marker categories, see Supplementary Table 2.

**Table 1:**
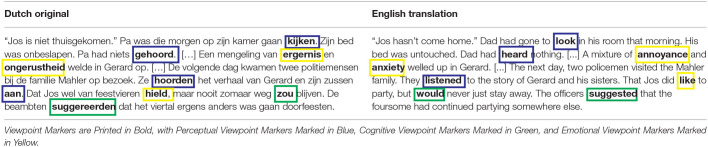
A coded excerpt from the stimulus narrative.

*Viewpoint Markers are Printed in Bold, with Perceptual Viewpoint Markers Marked in Blue, Cognitive Viewpoint Markers Marked in Green, and Emotional Viewpoint Markers Marked in Yellow*.

#### Measures of Social-Cognitive Abilities

As previous research is unclear about the specific aspects of social-cognitive abilities that could play a role in the processing of narrative viewpoint, we decided to use a combination of self-report and performance-based measures that tap into a broad spectrum of social-cognitive abilities. As much as possible, we included measures that were not susceptible to ceiling effects in a neurotypical population. Moreover, we included both linguistically-mediated tasks (e.g., Spontaneous Theory of Mind Protocol; Rice and Redcay, 2015) and measures that, at least at face value, are not linguistically-mediated (e.g., the emotional trials of the Multifaceted Empathy Test; Dziobek et al., 2008). We reasoned that if social-cognitive abilities, as measured with non-linguistic tasks, affect the processing of narrative viewpoint, this is extra strong evidence that there is a relationship between social cognition and narrative processing that goes beyond any potentially confounding effects of language ability.

##### Interpersonal Reactivity Index

As a first measure, we included the validated Interpersonal Reactivity Index (IRI; Davis, 1983), which is a multi-dimensional, self-report measure of trait empathy that taps into participants' tendency to feel concern for others (Empathic Concern, e.g., *I often have tender, concerned feelings for people less fortunate than me*), take the perspective of others (Perspective Taking, e.g., *I try to look at everybody's side of a disagreement before I make a decision*), feel anxious in emotional situations (Personal Distress, e.g., *I sometimes feel helpless when I am in the middle of a very emotional situation*), and emotionally engage with fictional others (Fantasy, e.g., *I really get involved with the feelings of the characters in a novel*). The 28 items of the IRI (Davis, 1983) were presented with 7-point scales (1 = *disagree*, 7 = *agree*). A Dutch translation partially based on De Corte et al. (2007) and Mak and Willems (2018) was used.

##### Multifaceted Empathy Test

Although previous research on the relationship between reading narratives and social-cognitive performance has often used the Reading the Mind in the Eyes Task (RMET; Baron-Cohen et al., 2001), this measure has recently received criticism for its poor internal consistency, homogeneity, and content validity (e.g., Olderbak et al., 2015; Turner and Felisberti, 2017; Black, 2019). Hence, as an alternative for the RMET we chose to include the Multifaceted Empathy Test (MET; Dziobek et al., 2008), which is a validated measure that uses participants' responses to ecologically valid pictures (i.e., full-body pictures of people in various daily situations experiencing a wide range of emotions) to assess both emotion recognition^3^ and emotional empathy. A potential downside of the MET is that it has been developed mainly for use in non-neurotypical populations (e.g., patients with an autism spectrum disorder, Dziobek et al., 2008; patients with narcissistic personality disorder, Ritter et al., 2011; patients with borderline personality disorder, Dziobek et al., 2011), and as a result may be susceptible to ceiling effects in a neurotypical population (Turner and Felisberti, 2017).

For the MET (Dziobek et al., 2008) participants viewed 40 pictures of people in various situations and were asked to select an emotion word from a list of four options that matched the emotion the person in each photo was experiencing as closely as possible (emotion recognition), and to rate the degree to which they “felt along” with the person in the picture by indicating the degree to which they experienced the same emotion as the person in the picture on a 9-point scale (1 = *not at all*, 9 = *a lot*; emotional empathy). Emotion recognition and emotional empathy were assessed in alternating blocks. Hence, each picture occurred twice: once in an emotion recognition block, and once in an emotional empathy block. Each block consisted of 10 pictures, resulting in a total of 8 blocks (4 emotion recognition blocks and 4 emotional empathy blocks). To avoid a confounding effect of vocabulary knowledge, a glossary of synonyms and example sentences for each emotion word that was used in the emotion recognition trials was provided.

The 109 German emotion words of the emotion recognition trials were translated into Dutch, using a similar method as Foell et al. (2018), who translated the test from German to English. The first author translated the words from German to Dutch using the online version of the dictionary *Van Dale Groot woordenboek der Nederlandse taal* (Den Boon and Geeraerts, 2005). Then, a backtranslation was performed by an independent German scholar. For 76 words, the backtranslation matched the original German word, in which case the Dutch translation was finalized. The procedure was repeated for the remaining 33 cases for which the backtranslation did not match the original German word. After the second round, 21 unclear cases remained. These were resolved by discussion between the first author and the German translator. The translation resulted in a list of 107 unique Dutch emotion words. In two cases, a single Dutch word was chosen as a translation for two distinct German words (*träumerisch* and *verträumt* were both translated as *dromerig*, “dreamy”; *beglückt* and *erfreut* were both translated as *verheugd*, “joyful”).

##### Visual Perspective-Taking Task

We also included the Visual Perspective-taking Task (VPT; Samson et al., 2010), which measures participants' ability to alternate between their own perspective and the perspective of an avatar. Although strictly speaking the VPT is a measure of visual perspective taking, we included it as a measure of social cognition, as the capacity to switch between egocentric and altercentric perspectives has been described as one of the fundamentals of social cognition (Fuchs, 2015). Moreover, aspects of trait empathy have been related to reduced altercentric intrusion, i.e., reduced interference from the perspective of the avatar (e.g., Nielsen et al., 2015; Mattan et al., 2016), supporting the use of the VPT as a measure of social cognition.

In the VPT (Samson et al., 2010), participants viewed 96 pictures of a room with an avatar in it and were asked to verify the number of circles visible on the side walls from either their own or the avatar's perspective. Before each picture was shown, a fixation cross appeared for 750 ms. After 500 ms, a cue appeared for 750 ms signaling participants to either verify their own perspective (YOU) or the perspective of the avatar (HE/SHE). 500 ms later, a number cue between 0 and 3 would appear for 750 ms. Lastly, the picture appeared on the screen. The participant's task was to verify whether the number cue matched the number of circles on the wall as visible from the perspective that was cued, i.e., their own perspective (YOU) or the perspective of the avatar (HE/SHE). Crucially, on half of the trials the number of circles visible from the participant's perspective was identical to the number of circles visible from the avatar's perspective (CONGRUENT), but on the other half of the trials a different number of circles would be visible from the different perspectives (INCONGRUENT). Participants used the mouse to indicate whether the number cue matched the number of circles seen from the cued perspective (MATCH; index finger) or not (MISMATCH; middle finger). If no answer was given within 2,000 ms, the next trial would start. Feedback was given after every trial. The pictures were presented in two blocks. Perspective, congruence, and correct response were counterbalanced. Six practice trials were presented at the start of the task, which could be repeated until the participant felt comfortable with the procedure.

##### Spontaneous Theory of Mind Protocol

Finally, the Spontaneous Theory of Mind Protocol (STOMP; Rice and Redcay, 2015) was included as a promising new measure that may be sensitive to individual variation among healthy adults (Rice and Redcay, 2015; Warnell and Redcay, 2019). Scores on this measure reflect a spontaneous tendency to mentalize when describing the events in two naturalistic videos and have been found to correlate with individual variability in cortical thickness of brain areas related to theory of mind in a neurotypical population (Rice and Redcay, 2015).

For the STOMP task (Rice and Redcay, 2015) participants viewed two silent videoclips taken from existing movies that are centered around complicated social interactions, and were then asked to describe what they had just seen in seven to ten sentences. One videoclip was a 2-min excerpt from the movie *John Tucker Must Die*, in which a girl comes back from a date with a boy whom she has to distract by pretending to flirt with him, so that her friend, who has been secretly following their date by hiding in his car, can escape. The other videoclip was a 3-min excerpt from the movie *Rear Window*, in which a woman is looking for something in an apartment, while being watched by the neighbors across the street, when the owner of the house comes home. Participants saw both videoclips in a random order.

#### Measures of Reading-Related Individual Differences

As we wanted to control for a possible confounding effect of print exposure, a Dutch version (Koopman, 2015) of the Author Recognition Test (ART; Stanovich and West, 1989) was used as an implicit measure of print exposure: participants were presented with a list of 30 real author names and 12 foils, and were asked to select the names of authors they knew.

Shallow narrative comprehension was measured using three multiple choice questions with four response options each (see Supplementary Materials) to check whether participants paid enough attention during reading. All participants scored above chance on these questions, hence, no data was excluded based on the comprehension questions.

### Data Recording and Stimulus Presentation

During reading, eye movements were recorded with a desktop-mounted EyeLink 1,000 plus eye tracker, recording at 1000 Hz. A head and chinrest were used to reduce head movements. For most participants the dominant eye was tracked, unless this lead to noisy signal, in which case the other eye was tracked (~15% of participants).

The narrative was presented using SR Research Experiment Builder. The narrative was divided into 56 sections that fit on the screen and contained between 42 and 151 words (*M* = 90.66, *SD* = 25.02). Most sections contained exactly one paragraph of the 64 paragraphs that made up the narrative, but in some cases the sections contained more than one paragraph, and/or a section break had to be inserted between sentences belonging to the same paragraph. The text was presented in black letters, set in 16 points Times New Roman, on a white page with 120 pixel margins on all sides and double line spacing on a BenQ XL 24020T 24″ LED screen (resolution: 1,024 × 768, 32 bits per pixel). Participants were seated 108 centimeters (42.52 inches) from the screen. The eye tracker was calibrated and validated on a 9-point grid until the largest difference between any target point and computed fixation position was <1°. A drift check and, if necessary, drift correction took place after every seven slides. At the start of each section a fixation cross marked the position of the first word for 1,000 ms. Participants used the space bar to go to the next section of the text. It was not possible to go back to a previous section.

All questionnaire-based measures (i.e., IRI, ART, and shallow comprehension) and the STOMP were administered digitally in Qualtrics (Provo, UT). The Multifaceted Empathy Test was presented with E-prime (version 2.0; Schneider et al., 2002), using the keyboard (numbers 1 through 9) to record responses. The Visual Perspective-taking Task was presented with DMDX (Forster and Forster, 2003), using a Logitech G502 HERO mouse with a polling frequency of 1,000 Hz to record reaction times.

### Procedure

The experiment took place in the Humanities Lab of Radboud University. Upon entering the lab, participants signed for informed consent. Then, participants filled in the IRI and ART questionnaire as well as two other questionnaires not relevant to the purposes of the current study on a laptop. After that, participants were tested on their eye dominance, and received instruction on the eye-tracking part of the experiment, which took place in a soundproof booth. After calibration of the eye tracker, participants read the narrative at their own pace while one eye was being tracked. After having finished reading, the participants completed the MET and VPT on the same computer in the soundproof booth. Then, the participants moved to the laptop outside the booth to complete the STOMP and the measure of shallow comprehension, as well as one other question not relevant for the purposes of the current study. Finally, participants were debriefed about the goal of the experiment and compensated for their time. Participants took between 60 to 90 min to complete the entire experiment. As described above, three participants were excluded because they were not able to finish the experiment within the available time.

### Data Analysis

#### Pre-processing of Eye-Tracking Data

Eye-tracking data were pre-processed in RStudio using popEye (Schroeder, 2019). PopEye is an R package that can be used to clean, pre-process, and analyze data from reading experiments. The default parsing algorithm from EyeLink was used for the parsing of fixations, saccades, and blinks. During the first stage of data pre-processing, fixations <80 ms were merged with any longer fixations within a 1-letter distance. In the second stage, fixations <40 ms were merged with any longer fixations within a 3-letter distance. Fixations that were more than 20% away from the text area were removed. Fixations were automatically aligned on the vertical axis to the lines of the text using the SpakovII algorithm (Špakov et al., 2019).

After the automatic pre-processing, all sections from all participants were inspected visually to check the quality of the automatic vertical alignment. If the automatic alignment of a section was incorrect because the underlying data were too noisy (e.g., horizontal drift) the section was rejected (i.e., removed from all analyses). If more than 30% of the sections of a participant had to be rejected, that participant was excluded. This led to the exclusion of 21 participants (see above). Of the remaining included participants, 317 sections (6.29%) had to be removed on this ground. If the automatic alignment of a section was incorrect but the quality of the underlying data was sufficient, the same pre-processing steps described above were applied again except this time outliers were not removed and vertical alignment was done manually. That is, fixations were visualized per section and for each sequence of fixations it was determined to which line the sequence belonged. This was done for 705 (13.99%) sections. For the remaining 4,018 (79.72%) sections, the automatically pre-processed and aligned data was of sufficient quality. After pre-processing, data from at least 40 of the original 56 sections was available for each participant (*M* = 52.48, *SD* = 4.14).

From the pre-processed data, eye-tracking measures were calculated. In line with recommendations by Kliegl and Laubrock (2017), Orquin and Holmqvist (2018), and von der Malsburg and Angele (2017), we decided against analyzing all of these measures, as this would greatly increase the risk of a Type-I error. Instead, we chose to focus on a small number of measures that covered both early and late processing: skip rate, gaze duration, and rereading rate. Skip rate, a binary variable that indicates whether a word has been fixated at any point during reading (skip rate = 0) or not (skip rate = 1), is usually associated with low-level word characteristics such as word length and word frequency (Inhoff and Radach, 1998; Brysbaert et al., 2005). However, it has also been found to be related to word predictability and context constraints (Brysbaert et al., 2005), making it an interesting candidate for our study, as viewpoint characteristics of words are both a lexical as well as a contextual phenomenon. Moreover, skip rate has been found to vary between readers (Faber et al., 2020), making it an interesting measure to detect individual differences.

Gaze duration reflects the total duration of fixations made on a word when it is first encountered and has been associated both with “later stages of word processing” (Radach and Kennedy, 2013, p. 431), as well as the “upper bound of early processing” (Kliegl and Laubrock, 2017, p. 77). As such, gaze durations might reflect the possible interaction between lexical characteristics (such as the viewpoint marker categories) and higher level processes (such as social-cognitive abilities). Moreover, gaze duration has often been found to be sensitive to individual differences between readers during narrative reading (van den Hoven et al., 2016; Mak and Willems, 2018; Vries et al., 2018; Eekhof et al., 2021).

Finally, rereading rate is a measure of late processing and reflects whether a word has been fixated again after the first run of reading (rereading rate = 1) or not (rereading rate = 0). The fact that this measure has been described as being relevant for cognitive processes that take place at the discourse level of texts (Rayner and Liversedge, 2011) makes it especially interesting for our study, as engaging with characters' viewpoints takes place at the discourse level as well.

In keeping with cautions expressed by Orquin and Holmqvist (2018), and Rayner and Liversedge (2011), we do not make direct qualitative assumptions about the connection between these eye-tracking measures and the exact linguistic or cognitive processes that they may reflect. However, in line with previous studies, we do assume that decreased skip rates and longer gaze durations reflect slower processing, potentially induced by processing difficulties (see e.g., Ashby et al., 2005; Rayner et al., 2011; Slattery and Yates, 2018; Gordon et al., 2020; Hessel and Schroeder, 2020). Rereading rate is relatively understudied, but Hessel and Schroeder (2020) found that words that were inconsistent with the context were reread more often, suggesting that increased rereading rate also reflects processing difficulties.

As a final cleaning step during pre-processing, gaze durations more than 3 standard deviations away from the subject-specific means were removed from all analyses. In addition, data from the first word of each section were removed from all analyses for each of the three measures. Function words were disregarded from all analyses, except function words that were part of a lexical unit that was coded as a viewpoint marker during application of the ViewPoint Identification Procedure. After data cleaning, content words had a mean skip rate of 0.27 (*SD* = 0.44), a mean gaze duration of 244.83 ms (*SD* = 103.42 ms), and a mean re-reading rate of 0.21 (*SD* = 0.41).

Because we wanted to control for possible confounding effects of word length and word frequency, all words were annotated for the number of letters and lemma frequency, taken from the SUBTLEX-NL corpus (Keuleers et al., 2010).

#### Statistical Analyses

Statistical analyses were performed in RStudio (RStudio version 1.3.959, R version 4.0.0; R Core Team, 2020). We calculated mean scores per participant for the four subscales of the IRI. Reliability was acceptable for all subscales (see Table 2), except the Empathic Concern subscale (α = 0.69). Consequently, the Empathic Concern subscale was not included in the analyses.

**Table 2:** Descriptive statistics for measures of social-cognitive abilities and reading-related individual differences.

**Measure**	***M* (*SD*)**	**Cronbach's α**
Interpersonal Reactivity Index—Perspective Taking	4.98 (0.85)	0.75
Interpersonal Reactivity Index—Personal Distress	3.57 (0.86)	0.74
Interpersonal Reactivity Index—Fantasy	5.04 (1.09)	0.85
Multifaceted Empathy Test—Emotional Empathy	5.02 (1.25)	0.95
Visual Perspective-taking Task—Altercentric Intrusion (ms)	28.80 (83.05)	
Visual Perspective-taking Task—Egocentric Intrusion (ms)	86.90 (85.20)	
Spontaneous Theory of Mind Protocol (%)	36.17 (10.04)	
Author Recognition Test	6.61 (3.28)	

*Interpersonal Reactivity Index scores could vary between 1 and 7 for all subscales, scores on the Multifaceted Empathy Test could vary between 1 and 9, Altercentric Intrusion (Visual Perspective-taking Task) varied between −279.71 and 269.69 ms, Egocentric Intrusion (Visual Perspective-taking Task) varied between −77.49 and 305.99 ms, scores on the Spontaneous Theory of Mind Protocol could vary between 0 and 100, and scores on the Author Recognition Test could vary between −12 and 30*.

ART scores were calculated by taking the number of correctly identified author names and subtracting the number of wrongly identified names (see Table 2).

Emotion recognition scores for the Multifaceted Empathy Test were calculated by adding up the number of correct answers per participant for the emotion recognition trials. However, reliability turned out to be unacceptable (α = 0.43). As reliability did not increase to be above 0.70 even after dropping half of the items, we decided to exclude this measure from the analyses. The reliability of the emotional empathy trials of the Multifaceted Empathy Test, on the other hand, was excellent (α = 0.95). Scores per participant were calculated by averaging over the 40 items (see Table 2).

In line with Samson et al. (2010), we only analyzed data from matching trials (i.e., trials in which the number of circles visible from the cued perspective matches the number cue) and correct trials (i.e., trials with incorrect responses were excluded) of the Visual Perspective-taking Task. Egocentric Intrusion was calculated by subtracting the mean response time for congruent other-trials from incongruent other-trials per participant. As such, the measure reflects the extra time needed to take up the altercentric perspective in the presence of a conflicting egocentric perspective, compared to when the altercentric perspective is congruent with the egocentric perspective. High scores on this measure thus indicate a poor ability to separate the two different perspectives and suppress the egocentric perspective in favor of the altercentric perspective. Altercentric Intrusion was calculated by subtracting the mean response time for congruent self-trials from incongruent self-trials per participant. As such, the measure reflects the extra time needed to take up the egocentric perspective in the presence of a conflicting altercentric perspective, compared to when the egocentric perspective is congruent with the altercentric perspective. High scores on this measure thus indicate a poor ability to separate the two different perspectives and suppress the altercentric perspective in favor of the egocentric perspective. Mean scores for both measures are reported in Table 2.

Participants' responses on the STOMP task were chunked by the first author based on Rice and Redcay's (2015) procedure of dividing sentences into clauses that represent individual units of information (for a full description of the chunking rules, see Supplementary Materials). These chunks were then coded by the first author as being either external descriptions (i.e., physical descriptions and descriptions of physical inferences) or internal descriptions (descriptions of emotions, intentions, and mental states), using a translated and enriched version of the original STOMP coding guide that contained definitions, examples, and key words for the two types of descriptions. An independent researcher then coded 20% of the data to assess the reliability of the coding. As inter-rater reliability was almost perfect (93.31%, κ = 0.86), the codes of the first author were used in further analyses. A STOMP score was calculated for each subject by taking the percentage of internal descriptions per subject. Seven participants indicated that they had seen one of the movies of which the excerpts were taken, in which case the STOMP score was only based on responses to the other excerpt. One participant had seen both movies, and as a result no STOMP score was calculated. Mean scores are reported in Table 2.

We used the *lme4* package (Bates et al., 2015) to fit linear mixed models for the continuous eye-tracking data (gaze duration) and generalized linear mixed models with a logit link function for the binary eye-tracking data (skip rate and rereading rate). In addition, we used the *lmerTest* package to estimate degrees of freedom and statistical significance for the linear mixed models (Kuznetsova et al., 2017). Variance Inflation Factors (VIFs) for the models were calculated with a function reported online (R-hack/mer-utils R, 2014). Predictors were scaled and centered for all analyses. In addition, lemma frequency was log-transformed. The eye-tracking data were analyzed at the word level. As the VPIP scores were available on the level of lexical units, these scores were transformed to the word level by giving all words belonging to a single lexical unit the same score.

We used an identical model structure for the analyses of skip rate, gaze duration, and rereading rate: all models included fixed effects of word length (continuous), word frequency (continuous), viewpoint marker category (factor with four levels: non-viewpoint marker, perceptual viewpoint marker, cognitive viewpoint marker, or emotional viewpoint marker), the measures of social cognition (the three IRI subscales, MET Emotional Empathy scores, Altercentric and Egocentric Intrusions taken from the VPT, and STOMP scores; all continuous), and ART scores (continuous), as well as interaction terms for the two-way interactions between viewpoint marker category and the measures of social cognition and between viewpoint marker category and ART scores. Finally, the models included by-subject random intercepts. Note that we did not add random slopes for viewpoint marker category, as this lead to convergence issues. Hence, the formula for the models was as follows:


$$\begin{array}{l} DV\;\sim \;word\;length+word\;frequency\\ +\;viewpoint\;marker\;category{\;}^{*}ART\;score\\ +\;viewpoint\;marker\;category{\;}^{*}IRI\;Perspective\;Taking\;score\\ +\;viewpoint\;marker\;category{\;}^{*}IRI\;Personal\;Distress\;score\\ +\;viewpoint\;marker\;category{\;}^{*}IRI\;Fantasy\;score\\ +\;viewpoint\;marker\;category{\;}^{*}STOMP\;score\\ +\;viewpoint\;marker\;category{\;}^{*}MET\;score\;\\ +\;viewpoint\;marker\;category{\;}^{*}Altercentric\;Intrusion\\ +\;viewpoint\;marker\;category{\;}^{*}Egocentric\;Intrusion\\ +\;\left(1|subject\right) \end{array}$$

We used dummy coding for the categorical predictor viewpoint markers category, using non-viewpoint markers as a reference level. Hence, for the main effect of viewpoint marker category, each level of viewpoint marker category (perceptual, cognitive, emotional) was compared to the non-viewpoint markers. With this type of contrast coding, the intercept represents the mean of the dependent variable for the reference level, i.e., non-viewpoint markers. Similarly, the estimates of the main effects of the other continuous predictors represent the effect estimate for the reference level, i.e., non-viewpoint markers. Estimates for the interactions between the other continuous predictors and the categorical variable viewpoint marker category indicate the difference between the estimate of the effect of the continuous variable for the reference level, i.e., non-viewpoint markers, and the estimate of the effect of the continuous variable for other levels of the categorical variable, i.e., the different categories of viewpoint markers.

The *sjPlots* package (version 2.8.7; Lüdecke, 2021) was used to produce output tables from the linear mixed models.

## Results

The descriptive statistics for the measures of social cognition and reading-related individual differences are given in Table 2. The descriptive statistics for the eye-tracking measures by viewpoint marker category are given in Table 3.

**Table 3:** Descriptive statistics for the eye-tracking data by viewpoint marker category.

	**Mean (** ***SD*** **)**			**Estimated marginal means (** ***SE*** **)**		
**Viewpoint marker category**	**Skip rate**	**Gaze duration**	**Re-reading rate**	**Skip rate**	**Gaze duration**	**Re-reading rate**
Non-viewpoint markers	0.27 (0.44)	244.67 (103.44)	0.21 (0.41)	0.24 (0.01)	242.04 (2.99)	0.19 (0.01)
Perceptual viewpoint markers	0.29 (0.45)	238.47 (100.73)	0.20 (0.40)	0.26 (0.01)	238.28 (3.25)	0.19 (0.01)
Cognitive viewpoint markers	0.21 (0.41)	244.59 (101.05)	0.21 (0.41)	0.19 (0.01)	247.38 (3.14)	0.20 (0.01)
Emotional viewpoint markers	0.16 (0.37)	259.72 (110.46)	0.25 (0.43)	0.22 (0.01)	246.76 (3.35)	0.22 (0.01)

### Skip Rate

The estimates for the generalized linear mixed model predicting skip rate are given in Table 4. VIFs were below 2 for all predictors. As expected, there was a significant relationship between the control variables word length and word frequency, and skip rate for non-viewpoint markers. An increase in word length decreased the odds of skipping by 0.45 times (i.e., long words were skipped less often) and an increase in word frequency increased the odds of skipping by 1.13 times (i.e., high-frequent words were skipped more often).

**Table 4:** Estimates for the generalized linear mixed model predicting skip rate.

**Predictors**	**Odds ratios**	***SE***	***CI***	***z***	***p***
(Intercept)	0.49	0.02	0.46–0.52	−20.61	<0.001^***^
Word length	0.45	0.00	0.44–0.46	−104.14	<0.001^***^
Word frequency	1.13	0.01	1.12–1.14	22.23	<0.001^***^
Viewpoint marker category (perceptual)	1.12	0.03	1.06–1.18	4.21	<0.001^***^
Viewpoint marker category (cognitive)	0.71	0.02	0.68–0.75	−14.09	<0.001^***^
Viewpoint marker category (emotional)	0.88	0.04	0.81–0.95	−3.16	0.002^**^
ART score	1.05	0.04	0.98–1.13	1.47	0.141
IRI—Perspective Taking score	1.08	0.04	1.00–1.17	2.05	0.041^*^
IRI—Personal Distress score	0.98	0.04	0.91–1.05	−0.56	0.573
IRI—Fantasy score	1.01	0.04	0.93–1.09	0.15	0.884
STOMP score	0.92	0.04	0.85–0.99	−2.18	0.029^*^
MET—Emotional Empathy score	0.96	0.04	0.89–1.05	−0.85	0.396
VPT—Altercentric Intrusion	1.01	0.04	0.94–1.09	0.33	0.741
VPT—Egocentric Intrusion	0.92	0.03	0.86–0.99	−2.23	0.026^*^
Viewpoint marker category (perceptual) ^*^ ART score	0.99	0.03	0.94–1.05	−0.34	0.737
Viewpoint marker category (cognitive) ^*^ ART score	1.04	0.02	0.99–1.09	1.62	0.106
Viewpoint marker category (emotional) ^*^ ART score	1.04	0.04	0.96–1.12	0.89	0.373
Viewpoint marker category (perceptual) ^*^ IRI—Perspective Taking score	1.07	0.03	1.01–1.14	2.21	0.027^*^
Viewpoint marker category (cognitive) ^*^ IRI—Perspective Taking score	0.99	0.03	0.94–1.05	−0.31	0.759
Viewpoint marker category (emotional) ^*^ IRI—Perspective Taking score	1.04	0.05	0.94–1.14	0.73	0.464
Viewpoint marker category (perceptual) ^*^ IRI—Personal Distress score	1.01	0.03	0.96–1.07	0.51	0.614
Viewpoint marker category (cognitive) ^*^ IRI—Personal Distress score	1.00	0.03	0.96–1.06	0.19	0.846
Viewpoint marker category (emotional) ^*^ IRI—Personal Distress score	1.01	0.04	0.93–1.10	0.29	0.773
Viewpoint marker category (perceptual) ^*^ IRI—Fantasy score	1.09	0.04	1.03–1.17	2.72	0.007^**^
Viewpoint marker category (cognitive) ^*^ IRI—Fantasy score	0.98	0.03	0.93–1.04	−0.68	0.494
Viewpoint marker category (emotional) ^*^ IRI—Fantasy score	1.04	0.05	0.94–1.14	0.70	0.484
Viewpoint marker category (perceptual) ^*^ STOMP score	1.01	0.03	0.95–1.07	0.19	0.850
Viewpoint marker category (cognitive) ^*^ STOMP score	1.02	0.03	0.97–1.08	0.82	0.414
Viewpoint marker category (emotional) ^*^ STOMP score	1.00	0.05	0.91–1.09	−0.07	0.947
Viewpoint marker category (perceptual) ^*^ MET—Emotional Empathy score	0.97	0.03	0.91–1.04	−0.85	0.395
Viewpoint marker category (cognitive) ^*^ MET—Emotional Empathy score	1.01	0.03	0.96–1.08	0.46	0.643
Viewpoint marker category (emotional) ^*^ MET—Emotional Empathy score	0.95	0.05	0.86–1.05	−1.02	0.308
Viewpoint marker category (perceptual) ^*^ VPT—Altercentric Intrusion	1.01	0.03	0.95–1.07	0.26	0.798
Viewpoint marker category (cognitive) ^*^ VPT—Altercentric Intrusion	0.94	0.02	0.90–0.99	−2.30	0.021^*^
Viewpoint marker category (emotional) ^*^ VPT—Altercentric Intrusion	1.04	0.05	0.96–1.14	0.98	0.327
Viewpoint marker category (perceptual) ^*^ VPT—Egocentric Intrusion	0.97	0.03	0.91–1.02	−1.24	0.214
Viewpoint marker category (cognitive) ^*^ VPT—Egocentric Intrusion	0.99	0.02	0.94–1.04	−0.53	0.597
Viewpoint marker category (emotional) ^*^ VPT—Egocentric Intrusion	0.92	0.04	0.84–1.00	−1.91	0.056

*All continuous predictors were scaled and centered for analysis. Word frequency was log-transformed for analysis. Dummy coding was used for the categorical predictor Viewpoint Marker Category with non-viewpoint markers as the reference level. Hence, for the main effect of viewpoint marker category, all categories were compared to non-viewpoint markers. The intercept represents the mean odds ratios of skipping for non-viewpoint markers. The estimates of the other main effects represent the effect estimate for non-viewpoint markers. The estimates for the interaction terms represent the difference between the effect estimate for non-viewpoint markers and the effect estimate for that specific viewpoint marker category*.

* *p < 0.05*,

** *p < 0.01*,

*** *p < 0.001*.

There was also a significant relationship between viewpoint marker category and skip rate (see also Table 3). Compared to non-viewpoint markers, the odds of skipping perceptual viewpoint markers were increased by 1.12 times compared to non-viewpoint markers (i.e., these markers were skipped more often). On the other hand, the odds of skipping cognitive and emotional viewpoint markers were decreased by 0.71 and 0.88 times, respectively (i.e., these markers were skipped less often).

In addition, there were also significant main effects of IRI Perspective Taking scores, STOMP scores, and Egocentric Intrusion on skip rate for non-viewpoint markers. An increase in IRI Perspective Taking scores increased the odds of skipping non-viewpoint markers by 1.08 times. That is, readers with higher self-reported perspective-taking abilities were more likely to skip non-viewpoint markers. On the other hand, an increase in STOMP scores decreased the odds of skipping non-viewpoint markers by 0.92 times. That is, readers with a higher tendency to spontaneously mentalize, were less likely to skip non-viewpoint markers. Finally, an increase in Egocentric Intrusion also decreased the odds of skipping non-viewpoint markers by 0.92 times. That is, readers with higher Egocentric Intrusion scores, i.e., poor visual perspective takers, were less likely to skip non-viewpoint markers.

Next, we inspected the interactions between specific viewpoint markers and predictors measuring social-cognitive abilities, to see whether there was a difference between the effect of social-cognitive abilities on non-viewpoint markers and the effect of these abilities on specific types of viewpoint markers. In other words, the interactions allowed us to see whether there was a specific effect of certain social-cognitive abilities on the processing of viewpoint markers that surpasses the effect of these abilities on non-viewpoint markers.

**Figure 1:**
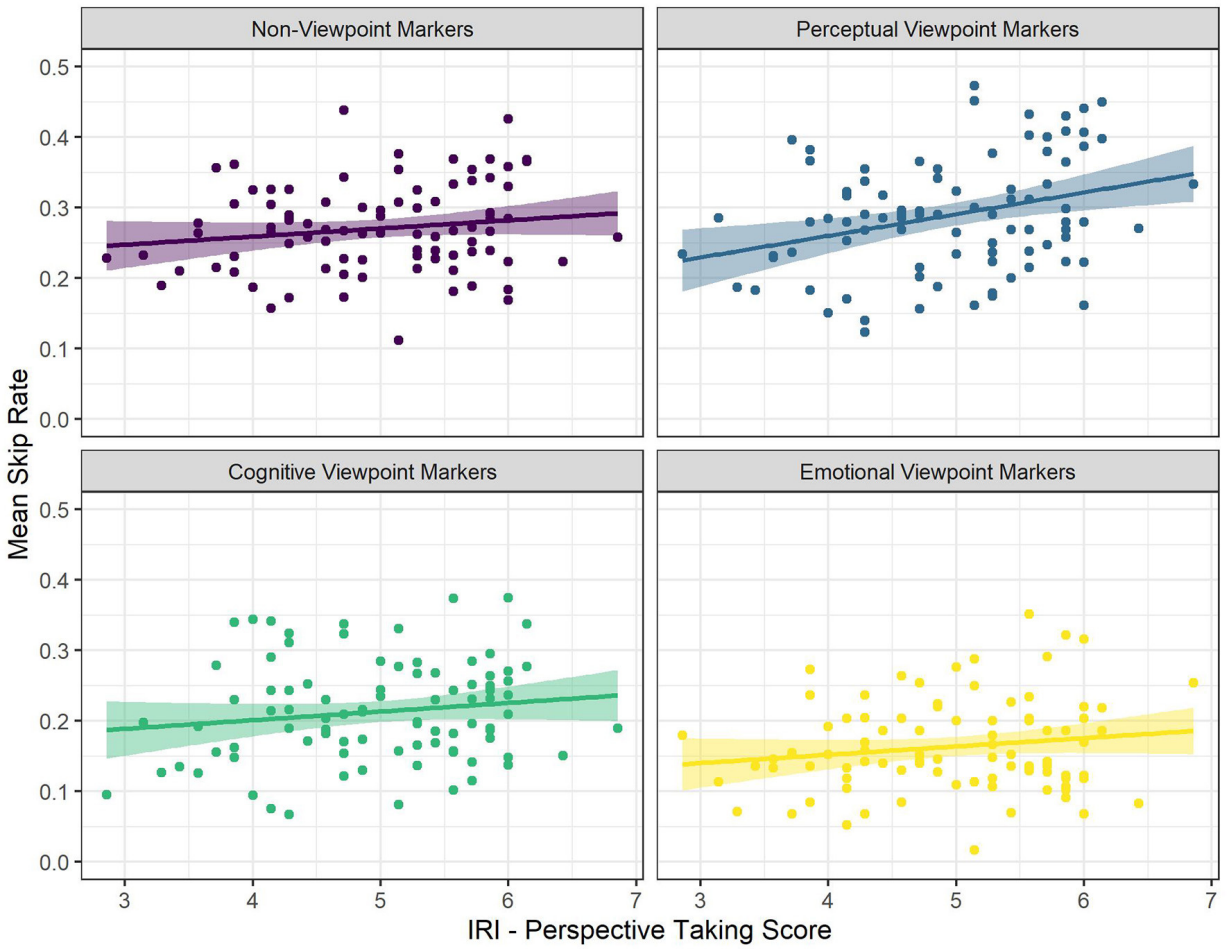
The Relationships Between Mean Skip Rate and IRI Perspective Taking Score for the Different Categories of Viewpoint Markers. Each Dot Represents a Participant.

**Figure 2:**
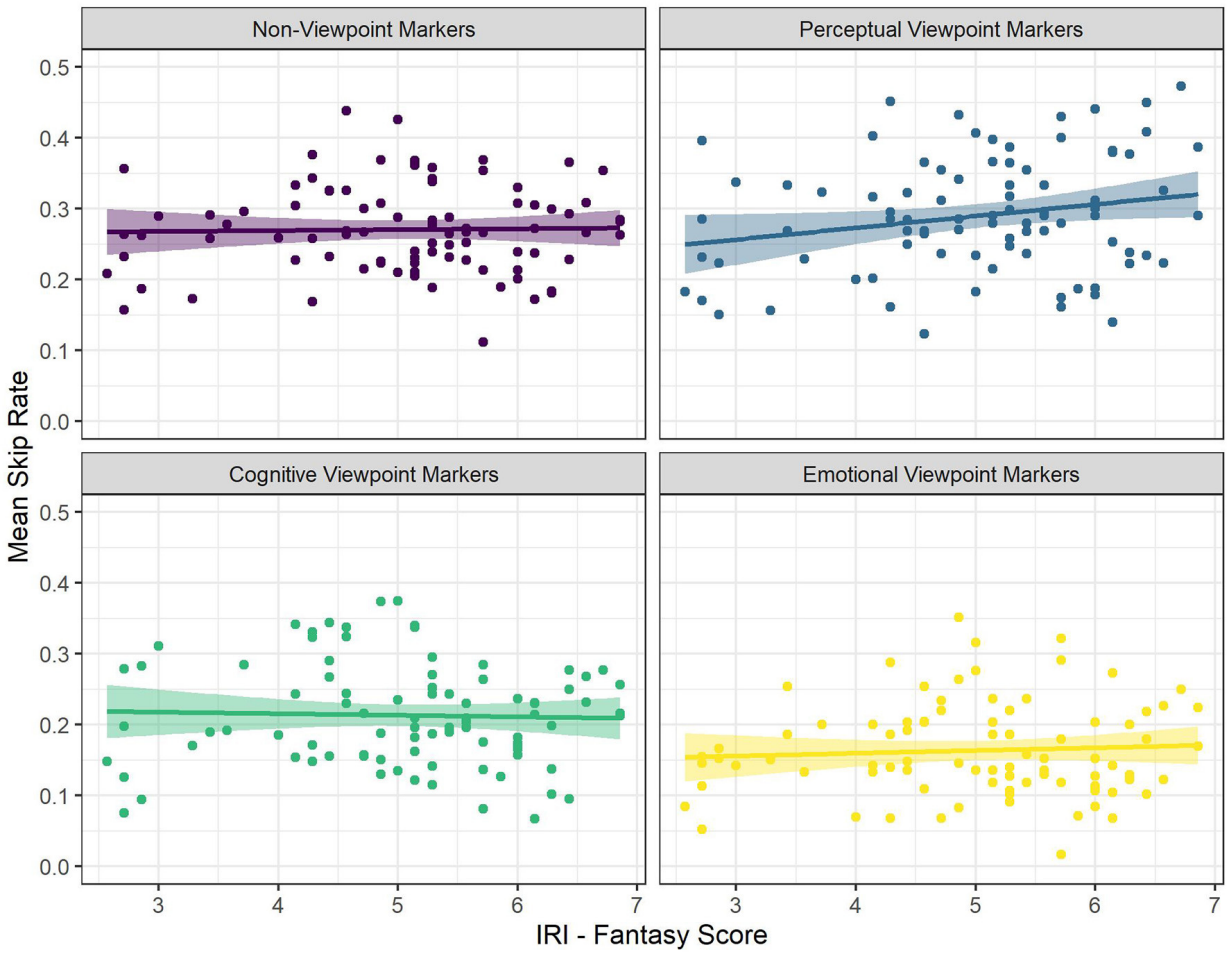
The Relationships Between Mean Skip Rate and IRI Fantasy Score for the Different Categories of Viewpoint Markers. Each Dot Represents a Participant.

There were significant interactions between viewpoint marker category (perceptual viewpoint markers) and both IRI Perspective Taking scores (see Figure 1) and IRI Fantasy scores (see Figure 2). There was a significantly more positive effect of both IRI Perspective Taking and IRI Fantasy scores on skip rate for perceptual viewpoint markers, compared to non-viewpoint markers. That is, for non-viewpoint markers, IRI Perspective Taking scores had a significantly positive effect and IRI Fantasy scores had a numerically positive, but non-significant effect on skip rate. For perceptual viewpoint markers, however, the effects of these scores were even more positive. In other words, for perceptual viewpoint markers the odds of skipping increased more as a result of being a reader with a high tendency to take the perspective of others than for non-viewpoint markers.

**Figure 3:**
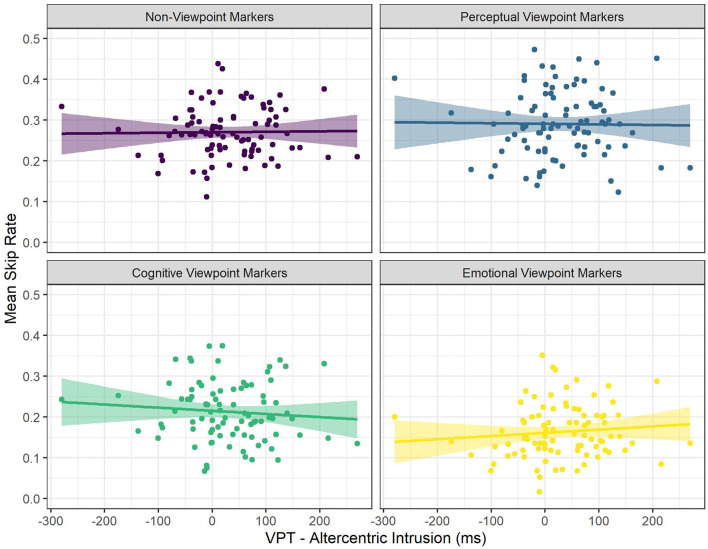
The Relationship Between Mean Skip Rate and Altercentric Intrusion for the Different Categories of Viewpoint Markers. Each Dot Represents a Participant.

In addition, there was a significant interaction between viewpoint marker category (cognitive viewpoint markers) and Altercentric Intrusion, such that there was a significantly more negative effect of Altercentric Intrusion on skip rate for cognitive viewpoint markers, compared to non-viewpoint markers (see Figure 3). That is, for non-viewpoint markers, Altercentric Intrusion had a numerically positive, but non-significant effect on skip rate, but for cognitive viewpoint markers the effect of Altercentric Intrusion was significantly more negative. In other words, for cognitive viewpoint markers the odds of skipping decreased more as a result of being an inflexible perspective taker than for non-viewpoint markers.

To follow up on these significant interactions, we ran two additional models on a subset of the data containing only perceptual viewpoint markers (for the interaction between perceptual viewpoint markers and the two IRI subscales) and a subset of the data containing only cognitive viewpoint markers (for the interaction between cognitive viewpoint markers and Altercentric Intrusion). The first follow-up model predicted skip rate for perceptual viewpoint markers with word length, word frequency, ART score, IRI Perspective Taking score and IRI Fantasy score as predictors, and by-subject random intercepts. This model confirmed that IRI Perspective Taking scores had a significant, positive effect on skip rate, such that an increase in IRI Perspective Taking scores increased the odds of skipping perceptual viewpoint markers by 1.13 times [*SE* = 0.05, *CI* (1.04–1.24), *z* = 2.74, *p* = 0.006; see Supplementary Table 3]. The effect of IRI Fantasy scores was not significant in this model [odds ratio = 1.07, *SE* = 0.05, *CI* (0.98–1.17), *z* = 1.52, *p* = 0.129; see Supplementary Table 3]. Hence, even though the effect of IRI Fantasy scores on skip rate for perceptual viewpoint markers differed significantly from the effect of IRI Fantasy scores on skip rate for non-viewpoint markers, there was by itself no significant effect of IRI Fantasy scores on skip rate when just looking at perceptual viewpoint markers. In other words, although the tendency to take the perspective of others did increase the odds of skipping perceptual viewpoint markers specifically, the tendency to fantasize did not.

The second follow-up model predicted skip rate for cognitive viewpoint markers with word length, word frequency, ART score, and Altercentric Intrusion as predictors, and by-subject random intercepts. This model revealed that there was in fact no significant effect of Altercentric Intrusion on skip rate for cognitive viewpoint markers [odds ratio = 0.95, *SE* = 0.05, *CI* (0.86–1.04), *z* = −1.14, *p* = 0.255; see Supplementary Table 4]. Hence, even though the effect of Altercentric Intrusion on skip rate for cognitive viewpoint markers differed significantly from the effect of Altercentric Intrusion on skip rate for non-viewpoint markers, there was by itself no significant effect of Altercentric Intrusion on skip rate when just looking at cognitive viewpoint markers. In other words, it was not the case that cognitive viewpoint markers specifically were skipped less often by readers who were poor visual perspective takers.

Note that even though there were significant effects of STOMP scores and Egocentric Intrusion on skip rate, there were no significant interactions between any of the viewpoint marker categories and Egocentric Intrusion. Hence, the effects of STOMP scores and Egocentric Intrusion on skip rate held for all content words, and was not specific to any category of viewpoint markers.

All in all, the results showed that perceptual viewpoint markers were skipped more often than non-viewpoint markers, whereas cognitive and emotional viewpoint markers were skipped less often than non-viewpoint markers. Furthermore, we found that STOMP scores and Egocentric Intrusion decreased the odds of skipping (i.e., readers with a high tendency to mentalize and poor visual perspective takers skip less often), but these effects were not specific to viewpoint markers. In addition, IRI Perspective Taking scores increased the odds of skipping (i.e., readers with high self-reported perspective-taking abilities skip more often) in general, and even more so for perceptual viewpoint markers in particular. That is, readers with higher IRI Perspective Taking scores were more likely to skip perceptual viewpoint markers, more so than non-viewpoint marking words in general. Although the effect of IRI Fantasy scores on skip rate was also significantly more positive for perceptual viewpoint markers than for non-viewpoint markers, a follow-up analysis revealed no significant main effect of IRI Fantasy scores on skip rate when just looking at perceptual viewpoint markers. Similarly, the effect of Altercentric Intrusion on skip rate was significantly more negative for cognitive viewpoint markers than for non-viewpoint markers, but a follow-up analysis revealed no significant main effect of Altercentric Intrusion on skip rate when just looking at cognitive viewpoint markers.

### Gaze Duration

The estimates for the linear mixed model predicting gaze duration are given in Table 5. VIFs were below 2 for all predictors. As expected, there was a significant relationship between the control variables word length and word frequency, and gaze duration for non-viewpoint markers, such that an increase in word length increased gaze duration (i.e., longer words were read slower) and an increase in word frequency decreased gaze duration (i.e., words with a higher frequency were read faster) for non-viewpoint markers.

**Table 5:** Estimates for the linear mixed model predicting gaze duration.

**Predictors**	**Estimates**	***SE***	***CI***	***t***	***p***
(Intercept)	230.78	2.99	224.91 – 236.65	77.11	<0.001^***^
Word length	11.41	0.28	10.86 – 11.96	40.53	<0.001^***^
Word frequency	−7.04	0.27	−7.56 – −6.51	−26.07	<0.001^***^
Viewpoint marker category (perceptual)	−3.88	1.33	−6.49 – −1.27	−2.91	0.004^**^
Viewpoint marker category (cognitive)	5.35	1.05	3.30 – 7.40	5.11	<0.001^***^
Viewpoint marker category (emotional)	4.79	1.56	1.74 – 7.85	3.08	0.002^**^
ART score	−10.70	3.04	−16.66 – −4.75	−3.52	0.001^***^
IRI—Perspective Taking score	−1.69	3.43	−8.40 – 5.03	−0.49	0.624
IRI—Personal Distress score	−0.91	3.11	−7.00 – 5.19	−0.29	0.771
IRI—Fantasy score	0.29	3.59	−6.74 – 7.33	0.08	0.935
STOMP score	−7.38	3.38	−14.00 – −0.75	−2.18	0.032^*^
MET—Emotional Empathy score	5.11	3.70	−2.14 – 12.36	1.38	0.170
VPT—Altercentric Intrusion	−0.74	3.07	−6.76 – 5.29	−0.24	0.811
VPT—Egocentric Intrusion	2.46	3.08	−3.58 – 8.49	0.80	0.427
Viewpoint marker category (perceptual) ^*^ ART score	2.07	1.35	−0.58 – 4.71	1.53	0.125
Viewpoint marker category (cognitive) ^*^ ART score	−0.66	1.05	−2.72 – 1.40	−0.63	0.530
Viewpoint marker category (emotional) ^*^ ART score	−1.78	1.56	−4.85 – 1.29	−1.14	0.256
Viewpoint marker category (perceptual) ^*^ IRI—Perspective Taking score	2.21	1.52	−0.78 – 5.20	1.45	0.147
Viewpoint marker category (cognitive) ^*^ IRI—Perspective Taking score	0.70	1.18	−1.61 – 3.00	0.59	0.554
Viewpoint marker category (emotional) ^*^ IRI—Perspective Taking score	1.91	1.79	−1.59 – 5.42	1.07	0.285
Viewpoint marker category (perceptual) ^*^ IRI—Personal Distress score	−1.66	1.38	−4.37 – 1.04	−1.21	0.228
Viewpoint marker category (cognitive) ^*^ IRI—Personal Distress score	−1.35	1.07	−3.45 – 0.75	−1.26	0.208
Viewpoint marker category (emotional) ^*^ IRI—Personal Distress score	−1.11	1.61	−4.27 – 2.04	−0.69	0.489
Viewpoint marker category (perceptual) ^*^ IRI—Fantasy score	−2.74	1.60	−5.88 – 0.39	−1.71	0.087
Viewpoint marker category (cognitive) ^*^ IRI—Fantasy score	−1.41	1.24	−3.84 – 1.02	−1.14	0.256
Viewpoint marker category (emotional) ^*^ IRI—Fantasy score	−4.90	1.87	−8.57 – −1.23	−2.62	0.009^**^
Viewpoint marker category (perceptual) ^*^ STOMP score	1.10	1.51	−1.86 – 4.05	0.73	0.468
Viewpoint marker category (cognitive) ^*^ STOMP score	−2.14	1.17	−4.44 – 0.16	−1.82	0.069
Viewpoint marker category (emotional) ^*^ STOMP score	−2.72	1.77	−6.19 – 0.74	−1.54	0.123
Viewpoint marker category (perceptual) ^*^ MET—Emotional Empathy score	−0.14	1.65	−3.38 – 3.10	−0.08	0.934
Viewpoint marker category (cognitive) ^*^ MET—Emotional Empathy score	1.47	1.29	−1.07 – 4.01	1.14	0.256
Viewpoint marker category (emotional) ^*^ MET—Emotional Empathy score	−1.83	1.95	−5.64 – 1.99	−0.94	0.348
Viewpoint marker category (perceptual) ^*^ VPT—Altercentric Intrusion	−0.32	1.39	−3.03 – 2.40	−0.23	0.819
Viewpoint marker category (cognitive) ^*^ VPT—Altercentric Intrusion	−0.93	1.09	−3.06 – 1.20	−0.86	0.391
Viewpoint marker category (emotional) ^*^ VPT—Altercentric Intrusion	−0.65	1.61	−3.81 – 2.50	−0.41	0.685
Viewpoint marker category (perceptual) ^*^ VPT—Egocentric Intrusion	2.09	1.36	−0.58 – 4.76	1.54	0.124
Viewpoint marker category (cognitive) ^*^ VPT—Egocentric Intrusion	−1.34	1.07	−3.43 – 0.75	−1.26	0.209
Viewpoint marker category (emotional) ^*^ VPT—Egocentric Intrusion	−1.49	1.58	−4.59 – 1.60	−0.95	0.344

*All continuous predictors were scaled and centered for analysis. Word frequency was log-transformed for analysis. Dummy coding was used for the categorical predictor viewpoint marker category with non-viewpoint markers as the reference level. Hence, for the main effect of viewpoint marker category, all categories were compared to non-viewpoint markers. The intercept represents the mean gaze duration for non-viewpoint markers. The estimates of the other main effects represent the effect estimate for non-viewpoint markers. The estimates for the interaction terms represent the difference between the effect estimate for non-viewpoint markers and the effect estimate for that specific viewpoint marker category*.

* *p < 0.05*,

** *p < 0.01*,

*** *p < 0.001*.

There was also a significant relationship between viewpoint marker category and gaze duration (see also Table 3). Compared to non-viewpoint markers, gaze durations were significantly decreased for perceptual viewpoint markers (i.e., faster reading), whereas gaze durations were significantly increased for cognitive and emotional viewpoint markers (i.e., slower reading).

In addition, there were also significant main effects of STOMP and ART scores on gaze durations for non-viewpoint markers. Both an increase in ART and STOMP scores decreased gaze durations. That is, readers with higher ART scores, indicative of print exposure, and readers with higher STOMP scores, indicative of a tendency toward spontaneous mentalizing, fixated non-viewpoint markers for a shorter duration.

Again, we inspected the interactions between specific viewpoint markers and predictors measuring social-cognitive abilities, to see whether there was a difference between the effect of social-cognitive abilities on non-viewpoint markers and the effect of these abilities on specific types of viewpoint markers. In other words, the interactions allowed us to see whether there was a specific effect of certain social-cognitive abilities on the processing of viewpoint markers that surpasses the effect of these abilities on non-viewpoint markers.

**Figure 4:**
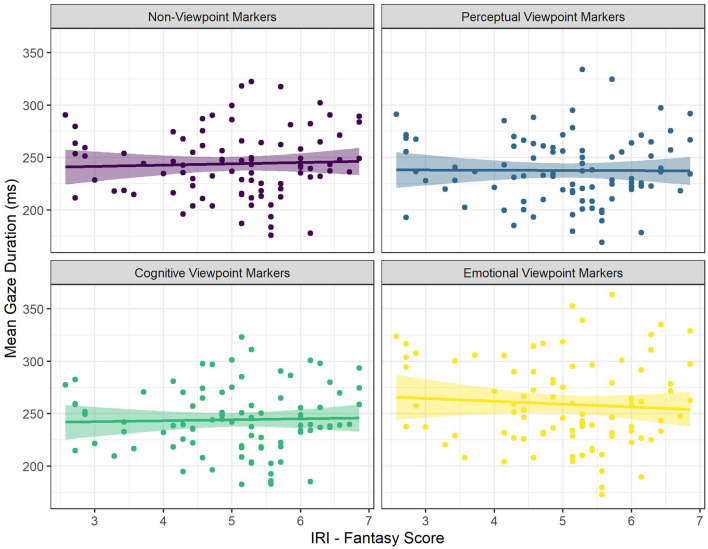
The Relationship Between Mean Gaze Duration and IRI Fantasy Score for the Different Categories of Viewpoint Markers. Each Dot Represents a Participant.

There was a significant interaction between viewpoint marker category (emotional viewpoint markers) and the Fantasy score of the IRI (see Figure 4). There was a significantly more negative effect of the Fantasy score on gaze duration for emotional viewpoint markers, compared to non-viewpoint markers. That is, for non-viewpoint markers the Fantasy score had a numerically positive, but non-significant effect on gaze duration, but for emotional viewpoint markers the effect of the Fantasy score was significantly more negative. In other words, for emotional viewpoint markers gaze durations decreased more as a result of being a reader with a high tendency to fantasize than for non-viewpoint markers.

To follow up on this significant interaction, we ran an additional model on a subset of the data containing only emotional viewpoint markers, predicting gaze duration for these markers with word length, word frequency, ART score, and IRI Fantasy score as predictors, and by-subject random intercepts. This model revealed that there was in fact no significant effect of IRI Fantasy score on gaze duration when just looking at emotional viewpoint markers [estimate = −3.19, *SE* = 4.17, *CI* (−11.36–4.97), *t* = −0.77, *p* = 0.443; see Supplementary Table 5]. Hence, even though the effect of the IRI Fantasy score on gaze duration for emotional viewpoint markers differed significantly from the effect of the IRI Fantasy score on gaze duration for non-viewpoint markers, there was by itself no significant effect of the IRI Fantasy score on gaze duration for emotional viewpoint markers. In other words, it was not the case that emotional viewpoint markers specifically were read faster by readers with higher IRI Fantasy scores.

Note that even though there were significant effects of STOMP and ART scores on gaze duration, there were no significant interactions between any of the viewpoint marker categories and these scores. Hence, the effect of STOMP and ART scores held for all content words, and was not specific to any category of viewpoint markers.

In sum, perceptual viewpoint markers were read relatively fast, whereas cognitive and emotional viewpoint markers were read relatively slow compared to non-viewpoint markers. In addition, we found that ART and STOMP scores decreased gaze durations overall. Although the effect of IRI Fantasy score on gaze duration was significantly more negative for emotional viewpoint markers compared to non-viewpoint markers, a follow-up analysis revealed that there was in fact no specific effect of IRI Fantasy scores on gaze duration when just looking at emotional viewpoint markers.

### Rereading Rate

The estimates for the generalized linear mixed model predicting rereading rate are given in Table 6. VIFs were below 2 for all predictors. As expected, there was a significant relationship between the control variables word length and word frequency, and rereading rate for non-viewpoint markers, such that an increase in word length increased the odds of rereading by 1.14 times (i.e., long words were reread more often) and an increase in word frequency decreased the odds of rereading by 0.93 times (i.e., high-frequent words were reread less often) for non-viewpoint markers.

**Table 6:** Estimates for the generalized linear mixed model predicting rereading rate.

**Predictors**	**Odds ratios**	***SE***	***CI***	***z***	***p***
(Intercept)	0.21	0.01	0.19–0.23	−36.63	<0.001^***^
Word length	1.14	0.01	1.12–1.15	18.25	<0.001^***^
Word frequency	0.93	0.01	0.91–0.94	−10.96	<0.001^***^
Viewpoint marker category (perceptual)	1.00	0.04	0.93–1.07	−0.10	0.921
Viewpoint marker category (cognitive)	1.07	0.03	1.01–1.13	2.40	0.017^*^
Viewpoint marker category (emotional)	1.16	0.04	1.08–1.25	3.89	<0.001^***^
IRI—Perspective Taking score	0.91	0.04	0.83–1.00	−1.95	0.051
IRI—Personal Distress score	0.97	0.04	0.89–1.05	−0.75	0.455
IRI—Fantasy score	0.97	0.05	0.88–1.07	−0.60	0.548
STOMP score	1.08	0.05	0.98–1.18	1.53	0.127
MET—Emotional Empathy score	1.08	0.06	0.97–1.20	1.47	0.141
VPT—Altercentric Intrusion	1.04	0.05	0.95–1.13	0.80	0.426
VPT—Egocentric Intrusion	1.09	0.05	1.00–1.18	1.90	0.057
ART score	1.10	0.05	1.01–1.20	2.28	0.023^*^
Viewpoint marker category (perceptual) * IRI—Perspective Taking score	1.01	0.04	0.93–1.10	0.29	0.769
Viewpoint marker category (cognitive) * IRI—Perspective Taking score	1.02	0.03	0.96–1.08	0.62	0.535
Viewpoint marker category (emotional) ^*^ IRI—Perspective Taking score	0.98	0.04	0.90–1.07	−0.42	0.672
Viewpoint marker category (perceptual) ^*^ IRI—Personal Distress score	0.98	0.04	0.91–1.05	−0.57	0.567
Viewpoint marker category (cognitive) ^*^ IRI—Personal Distress score	1.03	0.03	0.97–1.09	1.02	0.309
Viewpoint marker category (emotional) ^*^ IRI—Personal Distress score	1.02	0.04	0.94–1.10	0.48	0.630
Viewpoint marker category (perceptual) ^*^ IRI—Fantasy score	0.99	0.04	0.92–1.08	−0.13	0.899
Viewpoint marker category (cognitive) ^*^ IRI—Fantasy score	0.96	0.03	0.90–1.02	−1.28	0.199
Viewpoint marker category (emotional) ^*^ IRI—Fantasy score	1.02	0.05	0.93–1.12	0.48	0.633
Viewpoint marker category (perceptual) ^*^ STOMP score	0.97	0.04	0.90–1.05	−0.67	0.500
Viewpoint marker category (cognitive) ^*^ STOMP score	0.95	0.03	0.89–1.01	−1.65	0.098
Viewpoint marker category (emotional) ^*^ STOMP score	0.97	0.04	0.89–1.06	−0.60	0.549
Viewpoint marker category (perceptual) ^*^ MET—Emotional Empathy score	0.97	0.04	0.89–1.05	−0.81	0.417
Viewpoint marker category (cognitive) ^*^ MET—Emotional Empathy score	1.02	0.03	0.95–1.09	0.53	0.597
Viewpoint marker category (emotional) ^*^ MET—Emotional Empathy score	0.99	0.05	0.90–1.08	−0.31	0.755
Viewpoint marker category (perceptual) ^*^ VPT—Altercentric Intrusion	0.95	0.04	0.89–1.03	−1.28	0.199
Viewpoint marker category (cognitive) ^*^ VPT—Altercentric Intrusion	1.01	0.03	0.95–1.06	0.18	0.855
Viewpoint marker category (emotional) ^*^ VPT—Altercentric Intrusion	1.04	0.04	0.96–1.12	0.87	0.384
Viewpoint marker category (perceptual) ^*^ VPT—Egocentric Intrusion	1.00	0.03	0.93–1.07	−0.10	0.919
Viewpoint marker category (cognitive) ^*^ VPT—Egocentric Intrusion	1.07	0.03	1.01–1.13	2.42	0.016^*^
Viewpoint marker category (emotional) ^*^ VPT—Egocentric Intrusion	0.99	0.04	0.92–1.07	−0.29	0.768
Viewpoint marker category (perceptual) ^*^ ART score	1.01	0.04	0.94–1.08	0.26	0.794
Viewpoint marker category (cognitive) ^*^ ART score	1.04	0.03	0.98–1.09	1.31	0.191
Viewpoint marker category (emotional) ^*^ ART score	0.92	0.03	0.85–0.99	−2.32	0.020^*^

*All continuous predictors were scaled and centered for analysis. Word frequency was log-transformed for analysis. Dummy coding was used for the categorical predictor viewpoint marker category with non-viewpoint markers as the reference level. Hence, for the main effect of viewpoint marker category, all categories were compared to non-viewpoint markers. The intercept represents the mean odds ratios of rereading for non-viewpoint markers. The estimates of the other main effects represent the effect estimate for non-viewpoint markers. The estimates for the interaction terms represent the difference between the effect estimate for non-viewpoint markers and the effect estimate for that specific viewpoint marker category*.

* *p < 0.05*,

** *p < 0.01*,

*** *p < 0.001*.

There was also a significant relationship between viewpoint marker category and rereading rate (see Table 3). Compared to non-viewpoint markers, the odds of rereading cognitive and emotional viewpoint markers were increased by 1.07 and 1.16 times, respectively (i.e., these markers were reread more often). There was no significant effect of perceptual viewpoint markers on rereading rate compared to non-viewpoint markers.

In addition, there was also a significant main effect of ART scores on non-viewpoint markers. An increase in ART score increased the odds of rereading non-viewpoint markers by 1.10 times. That is, readers with higher ART scores, indicative of higher print exposure, reread non-viewpoint markers more often.

Again, we inspected the interactions between specific viewpoint markers and predictors measuring social-cognitive abilities, to see whether there was a difference between the effect of social-cognitive abilities on non-viewpoint markers and the effect of these abilities on specific types of viewpoint markers. In other words, the interactions allowed us to see whether there was a specific effect of certain social-cognitive abilities on the processing of viewpoint markers that surpasses the effect of these abilities on non-viewpoint markers.

**Figure 5:**
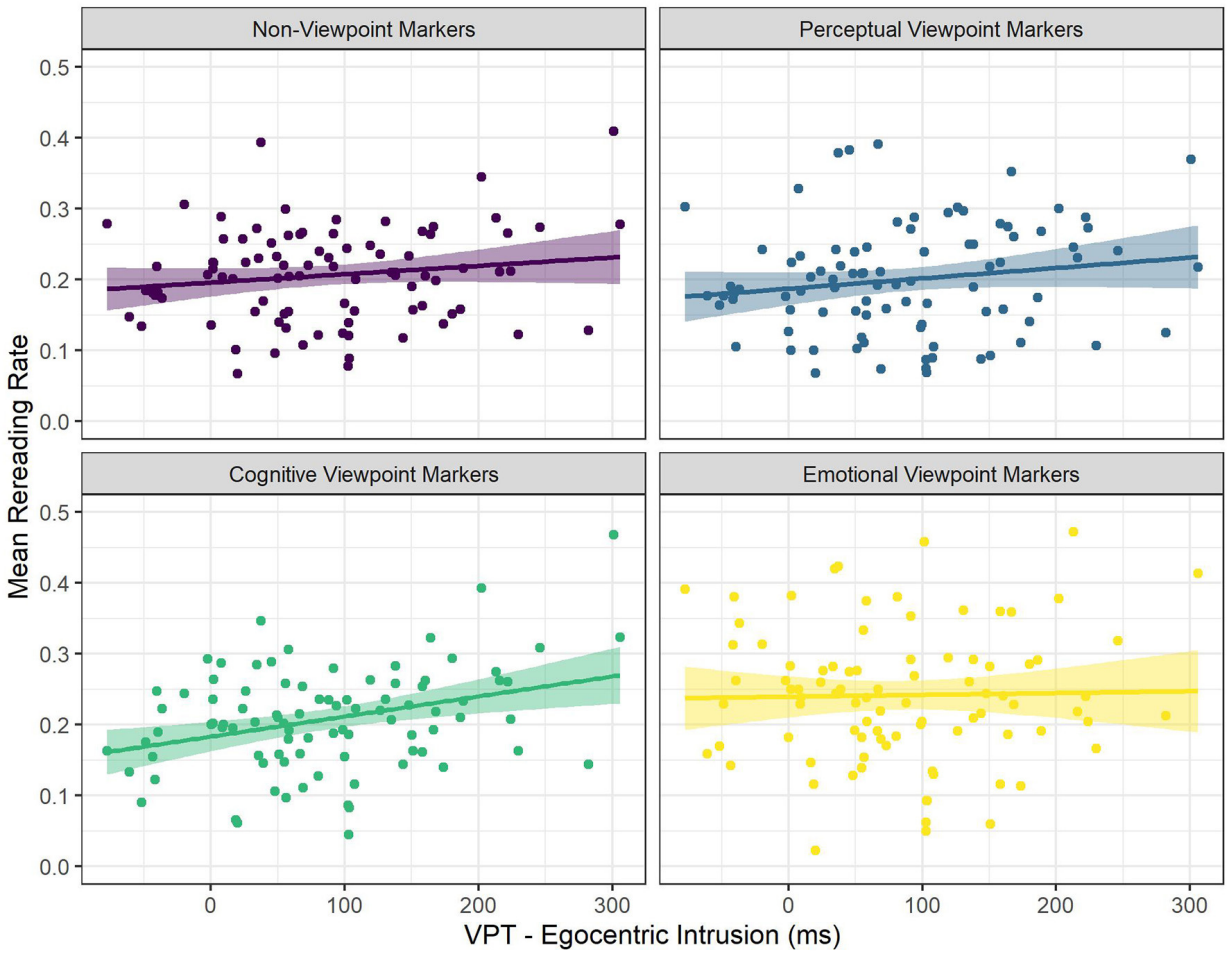
The Relationships Between Mean Rereading Rate and Egocentric Intrusion for the Different Categories of Viewpoint Markers. Each Dot Represents a Participant.

**Figure 6:**
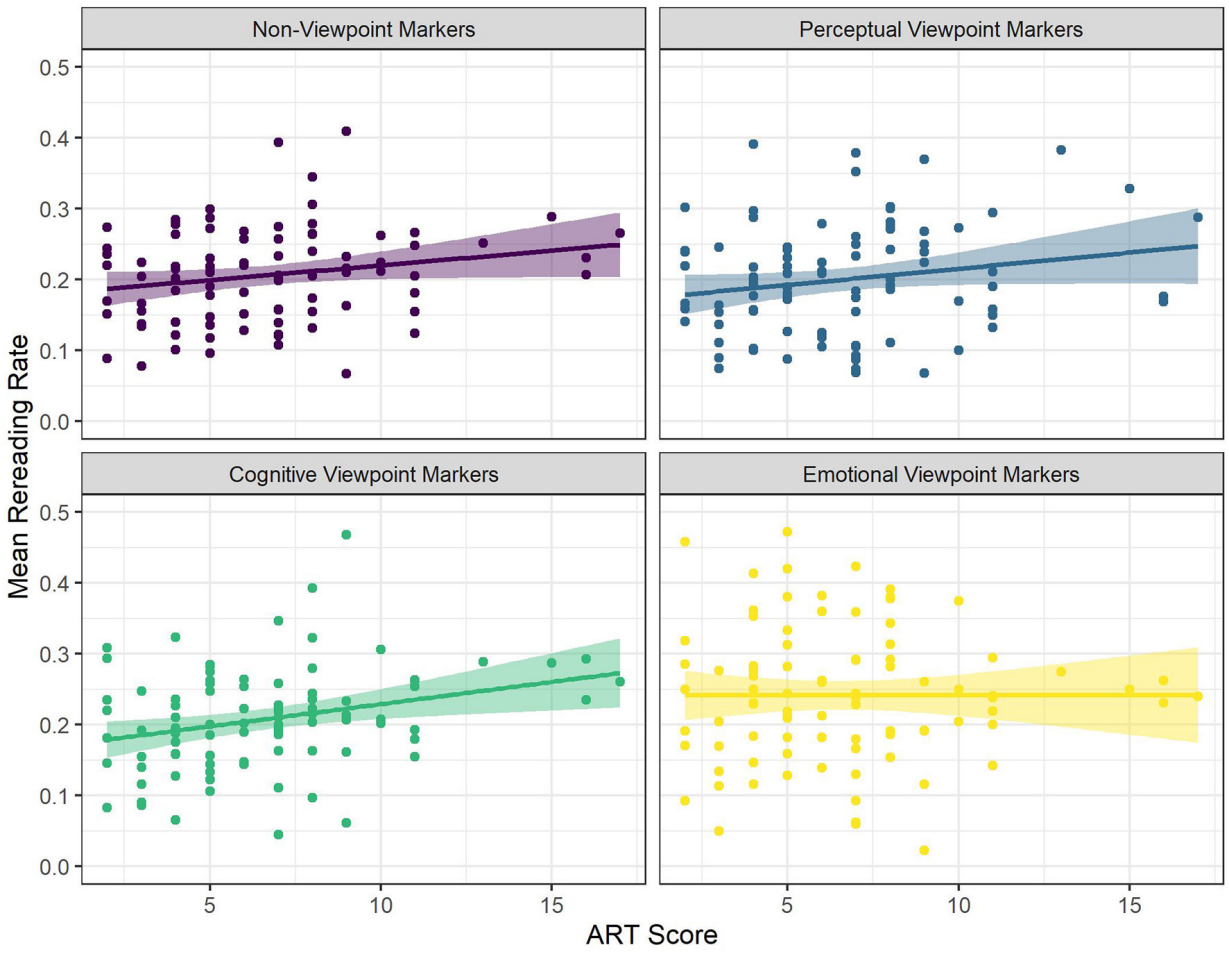
The Relationships Between Mean Rereading Rate and ART Score for the Different Categories of Viewpoint Markers. Each Dot Represents a Participant.

There were significant interactions between viewpoint marker category (cognitive viewpoint markers) and Egocentric Intrusion (see Figure 5), and between viewpoint marker category (emotional viewpoint markers) and ART scores (see Figure 6). There was a significantly more positive effect of Egocentric Intrusion on rereading rate for cognitive viewpoint markers, compared to non-viewpoint markers. That is, for non-viewpoint markers, Egocentric Intrusion had a numerically positive, near-significant effect on rereading rate, and this effect was significantly more positive for cognitive viewpoint markers. In other words, for cognitive viewpoint markers the odds of rereading increased more as a result of being a poor visual perspective taker than for non-viewpoint markers.

In addition, there was a significantly more negative effect of ART score on rereading rate for emotional viewpoint markers, compared to non-viewpoint markers. That is, for non-viewpoint markers, ART score had a significantly positive effect on rereading rate, but the effect of ART score was significantly more negative for emotional viewpoint markers, essentially meaning that contrary to non-viewpoint markers, rereading rate for emotional viewpoint markers was not affected by ART score.

To follow- up on the first significant interaction, we ran an additional model on a subset of the data containing only cognitive viewpoint markers. This model predicted rereading rate for cognitive viewpoint markers with word length, word frequency, ART score, and Egocentric Intrusions as predictors, and by-subject random intercepts. This model confirmed that Egocentric Intrusion had a significant, positive effect on rereading rate, such that an increase in Egocentric Intrusion increased the odds of rereading cognitive viewpoint markers by 1.14 times [*SE* = 0.05, *CI* (1.05–1.24), *z* = 3.03, *p* = 0.002; see Supplementary Table 6]. In other words, readers with poor visual perspective-taking abilities were more likely to reread cognitive viewpoint markers specifically.

To sum up, cognitive and emotional viewpoint markers were found to be reread more often than non-viewpoint markers, whereas perceptual viewpoint markers did not differ significantly from non-viewpoint markers. In addition, we found that ART score increased the odds of rereading (i.e., readers with higher print exposure reread more often), except for emotional viewpoint markers. Finally, Egocentric Intrusion increased the odds of rereading for cognitive viewpoint markers specifically (i.e., poor visual perspective takers reread cognitive viewpoint markers specifically more often).

**Figure 7:**
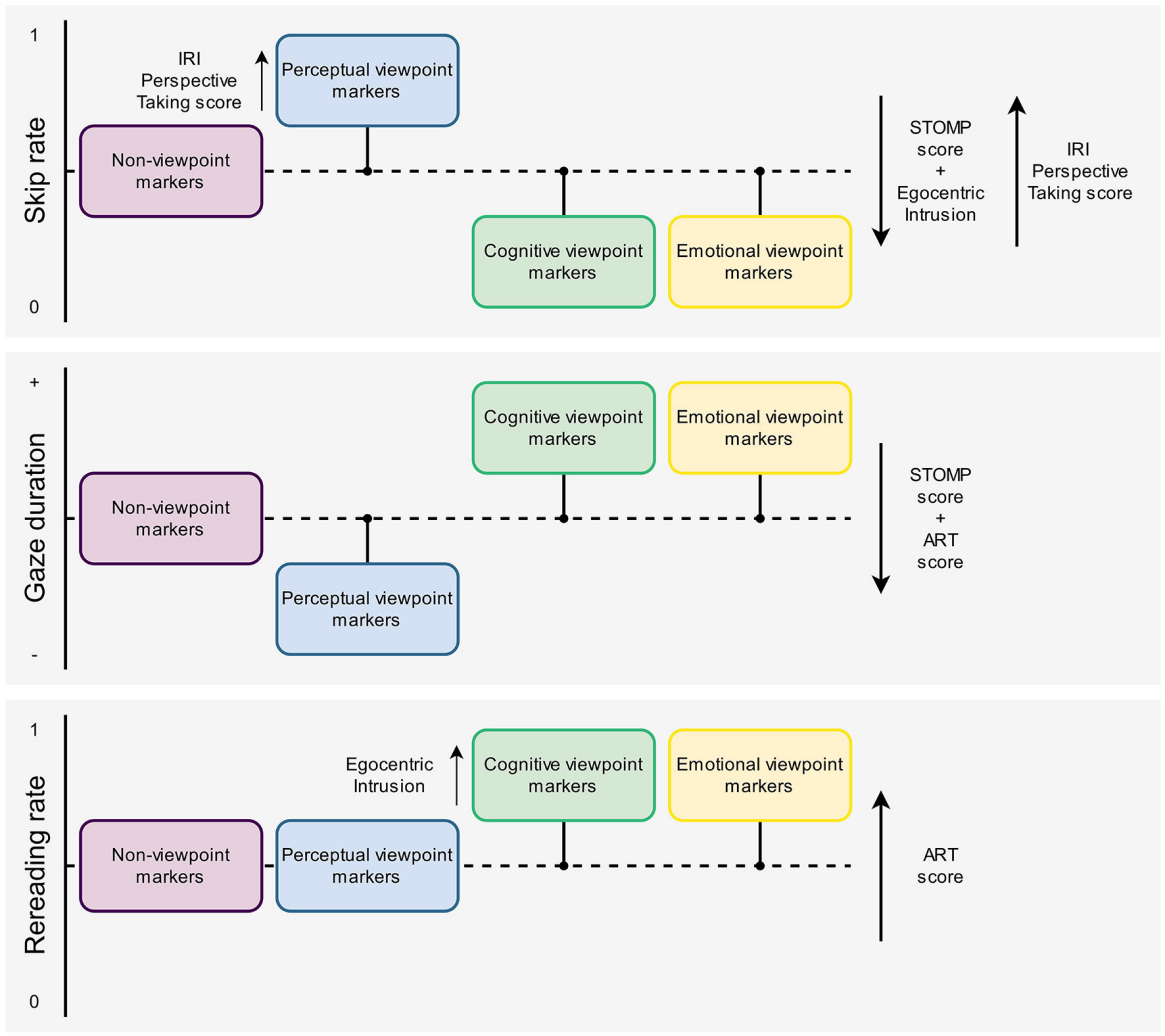
Schematic summary of the results: compared to non-viewpoint markers, perceptual viewpoint markers were skipped more often, whereas cognitive and emotional viewpoint markers were skipped less often. Egocentric Intrusion and STOMP score decreased skip rate overall, and IRI Perspective Taking score increased skip rate overall, and for perceptual viewpoint markers specifically. Compared to non-viewpoint markers, perceptual viewpoint markers were fixated shorter, whereas cognitive and emotional viewpoint markers were fixated longer. STOMP score and ART score decreased gaze durations overall. Compared to non-viewpoint markers, perceptual viewpoint markers did not differ in terms of rereading rate, whereas cognitive and emotional viewpoint markers were reread more often. ART score increased rereading rate overall, and Egocentric Intrusion increased rereading rate for cognitive viewpoint markers specifically.

The most important results are also schematically summarized in Figure 7.

## Discussion

In this article we set out to investigate the relationship between the processing of markers of narrative viewpoint, and social cognition. Specifically, we investigated how the linguistic processing of perceptual, cognitive, and emotional viewpoint markers during narrative reading is modulated by individual differences in social-cognitive abilities. We first looked at the effect of different types of viewpoint markers on eye movements and found diverging patterns of reading behavior for perceptual viewpoint markers on the one hand, and cognitive and emotional viewpoint markers on the other. Crucially, we also found that social-cognitive abilities modulated the effect of different viewpoint markers on eye movements, suggesting that the processing of narrative viewpoint engages these abilities during reading. In what follows, we will first discuss the differences in reading behavior for the three types of viewpoint markers. We will then focus on the role of social-cognitive abilities and the implications of our findings.

### Diverging Patterns of Reading Behavior for Different Viewpoint Dimensions

As expected, we found that cognitive and emotional viewpoint markers were skipped less, fixated longer, and reread more often compared to non-viewpoint marking content words. By contrast, however, perceptual viewpoint markers were fixated shorter and skipped more often than other non-viewpoint marking content words, and did not differ in terms of rereading rate from other non-viewpoint marking content words. In other words, whereas cognitive and emotional viewpoint markers were processed relatively slow, perceptual viewpoint marker were processed relatively fast compared to other content words, suggesting that the processing of perceptual narrative viewpoint is linguistically and/or conceptually simpler compared to the processing of cognitive and emotional narrative viewpoint (see also Mak and Willems, 2018).

When looking at the linguistic side of perceptual vs. cognitive and emotional perspective taking, it should first be noted that we controlled for differences in word length and word frequency in our analyses. Hence, the differences between perceptual viewpoint markers on the one hand, and cognitive and emotional viewpoint markers on the other, cannot be explained in terms of these basic linguistic characteristics. However, there might be additional semantic and syntactic differences between these viewpoint dimensions that could lead to differences in processing. For example, perceptual verbs such as *see* and *hear* are often classified as factive verbs (e.g., Givón, 1972), that is, expressing information that is assumed to be true, whereas most cognitive verbs such as *think* and emotional verbs such as *feel* are non-factives. Expressions of perception are thus one-dimensional in that they are implicative of the “truth” of what they express, while expressions of cognition and emotion are semantically multidimensional, referring to the speaker's stance toward the “truth” of what they express. Furthermore, in English, verbs of cognition have been found to be used with a sentential complement (*I think that it's raining*) more often than verbs of perception (*I see that it's raining*), which are more commonly used in simpler syntactic frames, such as in combination with direct objects (*I see rain*; Davis and Landau, 2020). As such, verbs of cognition and emotion might be semantically and syntactically more complex and thus take more time to process. In line with this hypothesis, Davis and Landau (2020) found that regardless of syntactic frame, children between 2 and 5 years old produced more verbs of perception (e.g., *to see, to hear*) than verbs of cognition (e.g., *to know, to think*). This finding is also in line with accounts of theory of mind and language acquisition that argue that children's perceptual understanding develops first, and subsequently serves as a model for understanding more abstract mental states such as beliefs (Gopnik et al., 1994).

Nonetheless, in our study, viewpoint markers were not only verbs, but also other types of content words. Another potential linguistic difference between the different types of viewpoint markers is therefore the distribution of word classes. For example, whereas the class of perceptual viewpoint markers contained mostly verbs, emotional viewpoint markers were rarely verbs and more often nouns and adjectives. However, perceptual and cognitive viewpoint markers were very similar in their proportion of different word classes, and yet differed in terms of reading behavior. All in all, more research is needed to understand how perceptual perspective taking, on the one hand, and cognitive and emotional perspective taking, on the other, differ, both linguistically and conceptually.

### The Role of Individual Differences in Social-Cognitive Abilities

Besides the differences in reading behavior for the different categories of viewpoint markers, we found that individual differences in social-cognitive abilities affected the processing of both words in general and, crucially, perceptual and cognitive viewpoint markers specifically. Firstly, we found that Egocentric Intrusion, a measure derived from the Visual Perspective-taking Task (Samson et al., 2010) that reflects the interference of one's own perspective when taking someone else's perspective, and scores on the Spontaneous Theory of Mind Protocol (Rice and Redcay, 2015), reflecting the spontaneous tendency to mentalize, decreased skip rate. That is, poorer perspective takers and readers with a high tendency to mentalize were less likely to skip words overall. In addition, scores on the Perspective Taking subscale of the Interpersonal Reactivity Index (Davis, 1983) increased skip rate, such that readers who are more likely to take the perspective of others, were more likely to skip words. Finally, scores on the Spontaneous Theory of Mind Protocol also decreased gaze durations for words overall, such that readers with high tendency to mentalize looked at words less long. Although the finding that STOMP scores decrease the odds of skipping words seems to contradict the other findings, the overall picture seems to be that readers with better social-cognitive abilities are faster readers (i.e., more skipping, shorter durations) in general.

The main aim of the study, however, was to see how social-cognitive abilities modulated the linguistic processing of viewpoint markers specifically. We found that readers with higher scores on the Perspective Taking subscale of the Interpersonal Reactivity Index were more likely to skip perceptual viewpoint markers in particular. Moreover, readers who experienced more egocentric intrusion and were thus less flexible perspective takers, were particularly more likely to reread cognitive viewpoint markers. These results cautiously suggest that besides a general facilitatory effect of social-cognitive abilities on linguistic processing, perspective-taking abilities facilitate the processing of at least perceptual and cognitive viewpoint markers. That is, the better these abilities (i.e., more self-reported perspective taking in daily life, more flexible visual perspective taking), the higher the likelihood that readers will skip perceptual viewpoint markers and not reread cognitive viewpoint markers.

What is puzzling, however, is why these two measures of perspective taking affect the processing of perceptual and cognitive viewpoint markers specifically, and not of all types of viewpoint markers. This might first and foremost be an issue of power: the stimulus narrative contained more than twice as many cognitive viewpoint markers as emotional viewpoint markers. Hence, future studies could look at more balanced narratives that contain an equal amount of perceptual, cognitive, and emotional viewpoint markers to see whether in such a case social-cognitive abilities affect the processing of all viewpoint dimensions. Alternatively, it could be the case that specific aspects of social-cognitive abilities are in fact related to specific types of narrative viewpoint processing. More detailed studies are needed to further elucidate the details behind these relationships.

All in all, our findings provide a first, modest piece of evidence that processing narrative viewpoint engages social-cognitive abilities, and that a weakness in these abilities thus leads to a delay in processing. As such, our findings corroborate earlier studies that have shown that social-cognitive abilities play a role in narrative processing (e.g., Pelletier and Wilde Astington, 2004; Mason and Just, 2009; Pavias et al., 2016; Atkinson et al., 2017). Moreover, we extend these findings by showing that these abilities are specifically related to the linguistic processing of narrative viewpoint, furthering our understanding of the exact aspects of narrative reading that social-cognitive abilities are implicated in. Our findings also resonate with developmental studies on the relationship between theory of mind and narrative comprehension in general (Pelletier and Wilde Astington, 2004; Pavias et al., 2016; Atkinson et al., 2017), and the acquisition and processing of epistemic markers, verbs of cognition, and verbs of emotion specifically (Moore et al., 1990; Antonietti et al., 2006; Grazzani and Ornaghi, 2012; Ornaghi and Grazzani, 2013). Interestingly, our study reveals that the relationship between social cognition and the processing of viewpoint markers such as verbs of cognition holds into adulthood.

A possible explanation for the finding that social-cognitive abilities facilitate the linguistic processing of narrative viewpoint could be that readers with high social-cognitive abilities have better linguistic or reading skills, for example because they read more often (Mar et al., 2006; Djikic et al., 2013; Mumper and Gerrig, 2017; Lenhart et al., 2020) or because social cognition (partially) relies on language, as is often argued for the relationship between language development and theory of mind development (e.g., Baird and Astington, 2005). Note, however, that we controlled for print exposure, as measured with the Author Recognition Test (Stanovich and West, 1989), in our analyses. Hence, there seems to be a unique contribution of social-cognitive abilities to the linguistic processing of viewpoint, independently of print exposure. To completely rule out this explanation, however, future research would benefit from including more measures of reading habits and skills.

Another conceivable explanation for the facilitatory effect of social-cognitive abilities could be that readers with high social-cognitive abilities process viewpoint markers faster because in light of these abilities viewpoint markers become (partially) redundant. That is, readers who can afford to do so might use their social-cognitive abilities to make sense of the viewpoints of characters, rather than depending too much on the linguistic cues that are provided in the text. In other words, these readers might use their social-cognitive abilities to decrease the demand on linguistic processing. On the other hand, readers with relatively poor social-cognitive skills might need to rely more on explicit markers of viewpoint, leading to slower linguistic processing. In other words, there might be trade-off between using social-cognitive or linguistic means to engage in narrative perspective taking.

This hypothesis is supported by a study on individual differences in perspective shifting: Duff (2018) found that, overall, readers were more likely to take the perspective of a character, rather than a narrator, when interpreting sentences that contained a verb of cognition (e.g., *know* or *believe*) compared to when the sentences contained no such predicate. However, this effect was found to interact with scores on the Autism Quotient questionnaire such that readers with high scores on this questionnaire were most sensitive to the presence of verbs of cognition. That is, unlike other participants, readers with high scores on the AQ took the perspective of the character almost exclusively when a verb of cognition was present. This suggests that readers with poor social-cognitive abilities are more sensitive to linguistic expressions of perspective.

The explanation that readers with better social-cognitive abilities rely less on textual cues such as viewpoint markers than readers with poor social-cognitive abilities also raises new questions. For example, how do readers with varying levels of social-cognitive abilities process narratives in which viewpoint markers are largely lacking and the contents of characters' minds has to be inferred based on external descriptions of behavior? If the proposed explanation holds, we would expect that readers with poor social-cognitive abilities would be impeded in their attempts to understand or identify with the story characters, because their ability to do so largely depends on explicit markers of viewpoint. On the other hand, readers with high social-cognitive skills would be able to compensate for the lack of explicit viewpoint marking with their social-cognitive abilities. An experiment in which the presence or absence of viewpoint markers is manipulated within narratives could be designed to test this hypothesis.

All in all our results suggest that linguistic markers of narrative viewpoint play a role in engaging social-cognitive abilities during reading. This finding is also of relevance for research on the positive effects of narratives on social cognition. If markers of narrative viewpoint engage social-cognitive abilities, then these abilities might be strengthened through repeated exposure to and engagement by narratives (Mar, 2018). Hence, markers of narrative viewpoint might be an interesting candidate in the search for textual determinants of the social-cognitive potential of narratives (see also Koopman and Hakemulder, 2015). Note that a recent study did not find a difference in the effect of a *single* exposure to a narrative with or without direct access to the inner worlds of protagonists on social-cognitive abilities (internal vs. external focalization; Wimmer et al., 2021). By contrast, reasoning from the present findings, it may be hypothesized that a study that combines such a textual approach with the individual differences approach outlined here, might reveal interesting patterns of results.

In conclusion, our experiment showed that individual differences in social cognition affect the linguistic processing of narratives, and specifically narrative viewpoint. Future research will need to further unravel what this means for narrative processes such as narrative empathy and identification, and, ultimately, the impact of narratives on social cognition.

## Data Availability Statement

The datasets presented in this study can be found in online repositories. The names of the repository/repositories and accession number(s) can be found at: www.osf.io/xdjtp.

## Ethics Statement

The studies involving human participants were reviewed and approved by Ethics Assessment Committee Humanities, Radboud University (approval code: 2018-3568). The participants provided their written informed consent to participate in this study.

## Author Contributions

LE, KK, JS, and RW conceived, designed the experiment, reviewed, edited, and wrote the final manuscript. LE collected and analyzed the data and drafted the first manuscript. All authors contributed to the article and approved the submitted version.

## Funding

This research was supported by a Veni Grant awarded to KK (Project Number 275-89-038) and a Vidi Grant awarded to RW (Project Number 276-89-007) from the Netherlands Organization for Scientific Research (NWO).

## Conflict of Interest

The authors declare that the research was conducted in the absence of any commercial or financial relationships that could be construed as a potential conflict of interest.

## Publisher's Note

All claims expressed in this article are solely those of the authors and do not necessarily represent those of their affiliated organizations, or those of the publisher, the editors and the reviewers. Any product that may be evaluated in this article, or claim that may be made by its manufacturer, is not guaranteed or endorsed by the publisher.

## Footnotes

^1^Source: Teunissen, P. (2015). *Zestien jaar vermist, zestien jaar zoeken. Waar was Joske gebleven?* Vrij Nederland. Available online at: https://www.vn.nl/zestien-jaar-vermist-zestien-jaar-zoeken-waar-was-joske-gebleven/.

^2^Due to experimenter error, 134 words of the original narrative were not presented to the participants. These words belonged to the introduction and were not crucial to the coherence or comprehensibility of the narrative plot structure.

^3^Although Dziobek et al. (2008) argue that the MET measures cognitive empathy, Oakley et al. (2016) have argued that social-cognitive tasks that measure participants' ability to assign mental states or emotions to pictures of faces or eyes reflect emotion recognition rather than theory of mind or cognitive empathy.

## Abbreviations

ART: Author Recognition Test
IRI: Interpersonal Reactivity Index
MET: Multifaceted Empathy Test
STOMP: Spontaneous Theory of Mind Protocol
VPT: Visual Perspective-taking Task.
