# Supplementary material for: Reading Minds, Reading Stories: Social-Cognitive Abilities Affect the Linguistic Processing of Narrative Viewpoint
Source: Front Psychol. 2021 Sep 28;12:698986. doi: 10.3389/fpsyg.2021.698986 (PMC8510643; doi:10.3389/fpsyg.2021.698986)
Supplement: Supplementary file 1 [file Table_1.docx]

**Supplementary Materials**

**Supplementary Table 1**

Examples of the Three Categories of Viewpoint Markers (Presented as Lemmas) from the Stimulus Narrative

| **Perceptual viewpoint markers** | **Cognitive viewpoint markers** | **Emotional viewpoint markers** |
| --- | --- | --- |
| Vinden (‘to find’) | Willen (‘to want’) | Voelen (‘to feel’) |
| Onherkenbaar (‘unrecognizable’) | Verwachten (‘to expect’) | Paniek (‘panic’) |
| Zien (‘to see’) | Poging (‘attempt’) | Uitkijken (‘to watch out’) |
| Loeren (‘to spy on’) | Eer (‘honour’) | Verdwaasd (‘foolish) |
| Blikken (‘looks’) | Voorstellen (‘to imagine’) | Grijns (‘grin’) |
| Horen (‘to hear’) | Moeten (‘to need to’) | Spannend (‘tense’) |
| Aanhoren (‘to listen to’) | Durven (‘to dare’) | Gek (‘mad’) |
| Bestuderen (‘to explore’) | Ondeugd (‘mischief’) | Geluk (‘happiness’) |
| Turen (‘to peer’) | Weten (‘to know’) | Radeloosheid (‘desperation’) |
| Getuige (‘witness’) | Sceptisch (‘sceptic’) | Verzoend (‘reconciled’) |
