# Supplementary material for: Reading Minds, Reading Stories: Social-Cognitive Abilities Affect the Linguistic Processing of Narrative Viewpoint
Source: Front Psychol. 2021 Sep 28;12:698986. doi: 10.3389/fpsyg.2021.698986 (PMC8510643; doi:10.3389/fpsyg.2021.698986)
Supplement: Supplementary file 2 [file Table_2.docx]

**Supplementary Table 2**

Contingency Table for the Relationship Between Word Class and Viewpoint Marker Category

|  |  | **Non-viewpoint markers** | **Perceptual viewpoint markers** | **Cognitive viewpoint markers** | **Emotional viewpoint markers** |
| --- | --- | --- | --- | --- | --- |
| Adjective | Count | 248 | 1 | 13 | 20 |
|  | % Within viewpoint marker category | 9.88 | 1.16 | 8.90 | 33.89 |
|  | Standardized residual | -0.30 | -2.60 | -0.44 | 5.77 |
| Adverb | Count | 321 | 0 | 1 | 0 |
|  | % Within viewpoint marker category | 12.79 | 0.00 | 0.69 | 0.00 |
|  | Standardized residual | 1.91 | -3.14 | -3.85 | -2.60 |
| Noun | Count | 997 | 3 | 10 | 20 |
|  | % Within viewpoint marker category | 39.72 | 3.49 | 6.85 | 33.90 |
|  | Standardized residual | 2.44 | -5.09 | -5.96 | -0.36 |
| Verb | Count | 847 | 82 | 117 | 18 |
|  | % Within viewpoint marker category | 33.75 | 95.35 | 80.14 | 30.51 |
|  | Standardized residual | -3.45 | 8.63 | 8.26 | -0.93 |
| NA | Count | 97 | 0 | 5 | 1 |
|  | % Within viewpoint marker category | 3.87 | 0.00 | 3.43 | 1.70 |
|  | Standardized residual | 0.49 | -1.78 | -0.16 | -0.79 |

*Note.* Word class information was taken from the SUBTLEX-NL corpus (Keuleers et al., 2010). Words from the stimulus narrative that were not in the corpus are marked as NA (not available). These mostly included names and proper nouns (e.g., *Joske*), complex compounds (e.g., *botsautomuntjes,* ‘bumper car coins’), and other uncommon words (e.g., *vermolmd*, ‘moldered’). A chi-square test of independence for the relation between word class and viewpoint marker category was significant: χ^2^(12) = 302.62, *p* < .001.
