# Supplementary material for: Reading Minds, Reading Stories: Social-Cognitive Abilities Affect the Linguistic Processing of Narrative Viewpoint
Source: Front Psychol. 2021 Sep 28;12:698986. doi: 10.3389/fpsyg.2021.698986 (PMC8510643; doi:10.3389/fpsyg.2021.698986)
Supplement: Supplementary file 3 [file Table_3.docx]

**Supplementary Table 3**

Estimates for the Generalized Linear Mixed Model Predicting Skip Rate for Perceptual Viewpoint Markers Only

| **Predictors** | **Odds ratios** | ***SE*** | ***CI*** | ***z*** | ***p*** |  |
| --- | --- | --- | --- | --- | --- | --- |
| (Intercept) | 0.56 | 0.02 | 0.51 – 0.61 | -13.06 | <0.001 | *** |
| Word length | 0.40 | 0.02 | 0.37 – 0.44 | -22.37 | <0.001 | *** |
| Word frequency | 1.03 | 0.05 | 0.93 – 1.13 | 0.50 | 0.618 |  |
| ART score | 1.05 | 0.05 | 0.97 – 1.15 | 1.21 | 0.227 |  |
| IRI – Perspective Taking score | 1.13 | 0.05 | 1.04 – 1.24 | 2.74 | 0.006 | ** |
| IRI – Fantasy score | 1.07 | 0.05 | 0.98 – 1.17 | 1.52 | 0.129 |  |

*Note*. All continuous predictors were scaled and centered for analysis. Word frequency was log-transformed for analysis.
* *p* < .05, ** *p* < .01, *** *p* < .001
