# Supplementary material for: Reading Minds, Reading Stories: Social-Cognitive Abilities Affect the Linguistic Processing of Narrative Viewpoint
Source: Front Psychol. 2021 Sep 28;12:698986. doi: 10.3389/fpsyg.2021.698986 (PMC8510643; doi:10.3389/fpsyg.2021.698986)
Supplement: Supplementary file 4 [file Table_4.docx]

**Supplementary Table 4**

Estimates for the Generalized Linear Mixed Model Predicting Skip Rate for Cognitive Viewpoint Markers Only

| **Predictors** | **Odds ratios** | ***SE*** | ***CI*** | ***z*** | ***p*** |  |
| --- | --- | --- | --- | --- | --- | --- |
| (Intercept) | 0.34 | 0.02 | 0.31 – 0.38 | -21.12 | <0.001 | *** |
| Word length | 0.46 | 0.02 | 0.43 – 0.50 | -19.11 | <0.001 | *** |
| Word frequency | 1.14 | 0.05 | 1.05 – 1.25 | 2.98 | 0.003 | ** |
| ART score | 1.11 | 0.05 | 1.01 – 1.23 | 2.20 | 0.028 | * |
| VPT – Altercentric Intrusion | 0.95 | 0.05 | 0.86 – 1.04 | -1.14 | 0.255 |  |

*Note*. All continuous predictors were scaled and centered for analysis. Word frequency was log-transformed for analysis.
* *p* < .05, ** *p* < .01, *** *p* < .001
