# Supplementary material for: Reading Minds, Reading Stories: Social-Cognitive Abilities Affect the Linguistic Processing of Narrative Viewpoint
Source: Front Psychol. 2021 Sep 28;12:698986. doi: 10.3389/fpsyg.2021.698986 (PMC8510643; doi:10.3389/fpsyg.2021.698986)
Supplement: Supplementary file 5 [file Table_5.docx]

**Supplementary Table 5**

Estimates for the Linear Mixed Model Predicting Gaze Duration for Emotional Viewpoint Markers Only

| **Predictors** | **Estimates** | ***SE*** | ***CI*** | ***t*** | ***p*** |  |
| --- | --- | --- | --- | --- | --- | --- |
| (Intercept) | 224.23 | 4.87 | 214.68 – 233.77 | 46.04 | <0.001 | *** |
| Word length | 13.45 | 2.03 | 9.47 – 17.43 | 6.63 | <0.001 | *** |
| Word frequency | -14.79 | 2.56 | -19.81 – -9.77 | -5.78 | <0.001 | *** |
| ART score | -12.82 | 4.16 | -20.97 – -4.66 | -3.08 | 0.002 | ** |
| IRI – Fantasy score | -3.19 | 4.17 | -11.36 – 4.97 | -0.77 | 0.443 |  |

*Note*. All continuous predictors were scaled and centered for analysis. Word frequency was log-transformed for analysis.
* *p* < .05, ** *p* < .01, *** *p* < .001
