# Supplementary material for: Reading Minds, Reading Stories: Social-Cognitive Abilities Affect the Linguistic Processing of Narrative Viewpoint
Source: Front Psychol. 2021 Sep 28;12:698986. doi: 10.3389/fpsyg.2021.698986 (PMC8510643; doi:10.3389/fpsyg.2021.698986)
Supplement: Supplementary file 6 [file Table_6.docx]

**Supplementary Table 6**

Estimates for the Generalized Linear Mixed Model Predicting Rereading Rate for Cognitive Viewpoint Markers Only

| **Predictors** | **Odds ratios** | ***SE*** | ***CI*** | ***z*** | ***p*** |  |
| --- | --- | --- | --- | --- | --- | --- |
| (Intercept) | 0.25 | 0.01 | 0.22 – 0.27 | -28.96 | <0.001 | *** |
| Word length | 1.03 | 0.04 | 0.96 – 1.11 | 0.92 | 0.357 |  |
| Word frequency | 1.06 | 0.05 | 0.98 – 1.16 | 1.47 | 0.141 |  |
| ART score | 1.14 | 0.05 | 1.04 – 1.24 | 2.96 | 0.003 | ** |
| VPT – Egocentric Intrusion | 1.14 | 0.05 | 1.05 – 1.24 | 3.03 | 0.002 | ** |

*Note*. All continuous predictors were scaled and centered for analysis. Word frequency was log-transformed for analysis.
* *p* < .05, ** *p* < .01, *** *p* < .001
